# Characteristics of Gut Microbiota in Patients With Rheumatoid Arthritis in Shanghai, China

**DOI:** 10.3389/fcimb.2019.00369

**Published:** 2019-10-23

**Authors:** Yang Sun, Qian Chen, Ping Lin, Rong Xu, Dongyi He, Weiqing Ji, Yanqin Bian, Yu Shen, Qingtian Li, Chang Liu, Ke Dong, Yi-Wei Tang, Zhiheng Pei, Liying Yang, Hongzhou Lu, Xiaokui Guo, Lianbo Xiao

**Affiliations:** ^1^Institute of Arthritis Research, Shanghai Academy of Chinese Medical Sciences, Shanghai, China; ^2^Guanghua Integrative Medicine Hospital, Shanghai, China; ^3^Department of Microbiology and Immunology, Shanghai Jiao Tong University School of Medicine, The College of Basic Medical Sciences, Shanghai, China; ^4^Clinical Laboratory, Shanghai Mental Health Center, Shanghai Jiao Tong University School of Medicine, Shanghai, China; ^5^Clinical Microbiology Service, Department of Laboratory Medicine, Memorial Sloan-Kettering Cancer Center, New York, NY, United States; ^6^Departments of Pathology and Medicine, New York University School of Medicine, New York, NY, United States; ^7^The Department of Veterans Affairs New York Harbor Healthcare System, New York, NY, United States; ^8^Department of Infectious Diseases, Shanghai Public Health Clinical Center, Fudan University, Shanghai, China

**Keywords:** rheumatoid arthritis, gut microbiome, 16S rRNA gene sequencing, inflammation, biomarker

## Abstract

Little is known regarding differences in the gut microbiomes of rheumatoid arthritis (RA) patients and healthy cohorts in China. This study aimed to identify differences in the fecal microbiomes of 66 Chinese patients with RA and 60 healthy Chinese controls. The V3-V4 variable regions of bacterial 16S rRNA genes were sequenced with the Illumina system to define the bacterial composition. The alpha-diversity index of the microbiome of the RA patients was significantly lower than that of the control group. The bacterial genera *Bacteroides* (*p* = 0.02202) and *Escherichia-Shigella* (*p* = 0.03137) were more abundant in RA patients. In contrast, *Lactobacillus* (*p* = 0.000014)*, Alloprevotella* (*p* = 0.0000008615)*, Enterobacter* (*p* = 0.000005759), and *Odoribacter* (*p* = 0.0000166) were less abundant in the RA group than in the control group. Spearman correlation analysis of blood physiological measures of RA showed that bacterial genera such as *Dorea* and *Ruminococcus* were positively correlated with RF-IgA and anti-CCP antibodies. Furthermore, *Alloprevotella* and *Parabacteroides* were positively correlated with the erythrocyte sedimentation rate, and *Prevotella*-2 and *Alloprevotella* were positively correlated with C-reactive protein, both biomarkers of inflammation. These findings suggest that the gut microbiota may contribute to RA development via interactions with the host immune system.

## Introduction

Rheumatoid arthritis (RA) is a common, chronic, autoimmune, and inflammatory disease affecting up to 1% of adults worldwide. It is characterized by progressive disability, systemic complications, early death, and socioeconomic costs (Firestein, 2003; Cooles and Isaacs, 2011). The pathogenesis of RA is complex, and the cause remains obscure.

Theories of the relationship between microbiome dysbiosis and RA date from the toxemic factor hypothesis originally proposed at the turn of the twentieth century. This hypothesis posited that the overgrowth of gram-negative bacteria in the intestines leads to an increase in toxic metabolites that enter the circulation and ultimately promote joint inflammation (Brusca et al., 2014). It is controversial whether RA is initiated by pathogenic bacteria (Tung et al., 1987; Scher and Abramson, 2011). Many bacteria have been proposed to be related to the morbidity of RA, including *Mycoplasma fermentans* (Sato et al., 2010), *Escherichia coli* (Newkirk et al., 2010), and *Proteus mirabilis* (Ebringer et al., 1985, 2010). *Mycbacterium avium* subspecies paratuberculosis (MAP), and mycobacterial antigens (Sharp et al., 2018), Some metabolites of these bacteria have been linked to RA. For instance, antibodies to *Proteus* urease (IRRET) and *Proteus* hemolysin (ESRRAL) might act as autoantibodies to hyaline cartilage (LRREI) and HLA-DR1/DR4 (EQRRAA), respectively (Ebringer and Rashid, 2014). Intriguingly, epidemiological and pathogenetic relationships between periodontitis and RA have emerged, mainly associated with *Porphyromonas gingivalis*, which has been associated with periodontitis, autoantibody formation, and joint inflammation (Mikuls et al., 2009; Hitchon et al., 2010; Scher and Abramson, 2011). In addition to certain infectious agents, intestinal commensal microbes may also be related to RA.

Trillions of bacteria inhabit the human body. Through various mechanisms, they interact with the physical nervous system, endocrine system, and immune system. Colonization with autoantigen ortholog-producing commensal bacteria may initiate and sustain chronic autoimmunity in genetically predisposed populations (Greiling et al., 2018). Notably, commensal bacteria appear to be important in suppressing the inflammatory response and promoting immunological tolerance. The microbiota can regulate the differentiation of lymphocyte subsets; moreover, the microbiota, or specific microbes, can affect immune functions. *Bacteroides fragilis* (Mazmanian et al., 2005; Round and Mazmanian, 2010) and *Faecalibacterium prausnitzii* (Sokol et al., 2008), which exist in the human intestine as members of the commensal microbiota, induce CD4^+^ T cells that secrete interleukin-10 (IL-10). Moreover, polysaccharide A of *B. fragilis* promotes immunological tolerance by activating Foxp3^+^ regulatory T cells and suppresses T helper 17 (Th17) cell responses via a Toll-like receptor-2-dependent pathway (Round et al., 2011). The colonization of the small intestines of mice with segmented filamentous bacteria (SFB) induces the appearance of CD4^+^ T helper cells, promotes the production of Th17 cells, and induces autoimmune diseases, such as arthritis, in susceptible mice (Ivanov et al., 2009). SFB colonization of the intestinal epithelium induces the expression of serum amyloid A (SAA) protein, which in turn stimulates lamina propria dendritic cells (DCs) to produce IL-6 and IL-23, thus inducing the differentiation of Th17 cells (Ivanov et al., 2009). Notably, SAA has been shown to play a role in chronic inflammation in patients with RA (Chambers et al., 1983), indicating that SFB may contribute to the interplay among the microbiome, Th17 cells, and autoimmune function. In addition, *Lactobacillus* is a probiotic genus of bacteria that reside in the intestine as commensal microbes and exert immunomodulatory functions.

Perturbation of the gut microbiome contributes to dysbiosis and leads to diseases, including immune dysfunction. The pathogenesis of autoimmune diseases is associated with the absence of tolerance checkpoints that normally prevent self-antigens from stimulating the relentless growth of self-reactive B and T lymphocytes (Goodnow, 2007). CD4+CD25+ regulatory T cells are crucial in regulating self-reactive T cells and preventing autoimmune disease (Kojima et al., 1976; Tung et al., 1987). The Single Nucleotide Polymorphisms (SNPs) in the negative regulators Protein Tyrosine Phosphatase Non-receptor type 2 and 22 (PTPN2/22) may lead a dysregulated immune response, and triggers continuing apoptosis in chronic inflammation in RA (Sharp et al., 2018). Recently, it has been reported that the single nucleotide polymorphisms (SNPs) of STAT4, PTPN2, PSORS1C1, and TRAF3IP2RA genes are associated with the clinical efficacy of tumor necrosis factor (TNF) inhibitors in the treatment of rheumatoid arthritis (RA) patients (Yang et al., 2018). Meanwhile, the activation of self-reactive CD4+ T cells is necessary, although not sufficient, for inducing autoimmune diseases. HLA-II molecules, especially in activating CD4+ T cells, are critical in triggering human immune response (Huang et al., 2018). Therefore, the regulation of T cell subsets is crucial in maintaining immune balance. The disturbed gut microbiome may also promote the expression of IL-23. Spore-forming species, especially *Clostridium clusters IV* and *XIVa*, enhance the expression of transforming growth factor-beta (TGF-β), indoleamine 2,3-dioxygenase, and Foxp3 and have been implicated in the induction or accumulation of regulatory T cells (Tregs) in the colonic mucosa (Atarashi et al., 2011; Chiba and Seno, 2011). TGF-β can increases CD39 expression on Tregs via the activation of TGFBRII/TGFBRI, SMAD2, and the transcription factor CREB, which is activated in a p38-dependent manner and induces CD39 expression by promoting ENTPD1 gene transcription (Peres et al., 2018). Regulatory T (Treg) cells which play a key role in the modulation of immune responses have an impaired function in RA. Foxp3 is a master regulator of Treg cells which its expression is under the tight control of epigenetic mechanisms (Parisa et al., 2018).

Many studies in the global community have focused on the connection between the gut microbiome and RA in recent years. These have revealed the depletion of *Bifidobacteria*, the *Bacteroides*-*Porphyromonas*-*Prevotella* group, the *Bacteroides fragilis* subgroup, and the *Eubacterium rectale*-*Clostridium coccoides* group in the microbiome of RA patients compared with that in patients with non-inflammatory fibromyalgia (Vaahtovuo et al., 2008). In addition, *Lactobacillus* communities are more abundant in early RA (Liu et al., 2013). However, there is limited knowledge of the differences in the gut microbiomes of RA patients and healthy cohorts in China. In this study, we recruited 126 individuals for analysis of the microbial composition in RA patients in order to identify a feasible diagnostic strategy for RA based on the fecal microbiome.

## Materials and Methods

### Ethics Statement

This study was approved by the Institutional Review Board (IRB) at Shanghai GuangHua Hospital of Integrated Traditional Chinese and Western Medicine. All participants gave written informed consent in line with the Declaration of Helsinki.

### Recruitment of Subjects and Sample Collection

Sixty-six patients with RA were recruited from Guanghua Hospital in Shanghai, China. Sixty healthy individuals also agreed to participate. RA patients fulfilled the RA criteria of the American College of Rheumatology. Fecal samples were obtained from all study subjects. All samples were transported in liquid nitrogen and stored at −80°C until the extraction of bacterial DNA.

### DNA Extraction and Amplification of V3-V4 Regions

Total DNA was extracted from fecal samples using the QIAamp DNA Stool Mini Kit (Qiagen, Duesseldorf, Germany) following the manufacturer's instructions (QIA amp DNA Stool Handbook 04/2010). The 16S rRNA high-throughput sequencing was performed by Majorbio (Shanghai, China) with the MiSeq instrument (Illumina Inc., San Diego, CA, USA). Variable regions V3-V4 of the 16S rRNA genes of bacteria were amplified with barcode-indexed primers 338F and 806R. The forward primer sequence was 5′-ACTCCTACGGGAGGCAGCA-3′, and the reverse primer sequence was 5′-GGACTACHVGGGTWTCTAAT-3′.

### MiSeq Sequencing

Amplicons were purified by 2% gel extraction (AxyPrep DNA Gel Extraction Kit, Axygen Biosciences, Union City, CA, USA) according to the manufacturer's instructions. The concentrations of polymerase chain reaction (PCR) products were measured using QuantiFluor^TM^-ST (Promega, Madison, WI, USA). Products were normalized at equimolar concentrations and subjected to paired-end sequencing (2 × 250) on the Illumina MiSeq platform (Illumina Inc.) according to standard protocols.

### OTU Clustering and Annotation

After sequencing, the raw paired-end reads with overlapping nucleotides were assembled by pandaseq. Subsequently, the reads were quality-filtered. OTUs were determined at a 97% similarity cut-off value, and clustering analysis was then conducted using Usearch (version 7.0, http://drive5.com/uparse/). The Silva database (Release128, http://www.arb-silva.de) was used to obtain specific taxonomic information corresponding to each OTU.

### Fecal Microbiome Analysis

The α-diversity was measured by species richness from the rarefied OTU table, and β-diversity was estimated by computational analysis using unweighted UniFrac scores and was visualized with principal coordinate analysis (Tang et al., 2018). Hierarchical cluster analysis was performed with single-linkage, complete-linkage, and average-linkage clustering methods. The LEfSe was used to elucidate differences in bacterial taxa. An LDA score ≥ 2 indicated that the bacterial group was an important contributor to RA. LEfSe analysis was performed online in the Galaxy workflow framework (http://huttenhower.sph.harvard.edu/galaxy/root?tool_id=lefse_upload).

### Quantitative Analysis of 16S rRNA Genes

Quantitative PCR (qPCR) was validated by constructing an artificial mixture of 16S rRNA genes according to published approaches (Becker et al., 2000; Bacchetti De Gregoris et al., 2011). The forward primer (5′-CATGTGGTTTAATTCGATGAT-3′) and the reverse primer (5′-AGCTGACGACAACCATGCAG-3′) were designed to detect *Bacteroides*.

### Statistical Analyses

All statistical analyses were performed using the R package (v. 3.2.2). Student's *t*-tests were performed to assess differences in the α-diversity of the two groups. For the comparison of continuous variables, the Mann-Whitney *U*-test was conducted for two groups. Multiple test correction was performed using the Benjamini and Hochberg false discovery rate. Spearman's rank test was performed for correlation analysis.

## Results

### Sample Size and Sequencing Depth

**Figure 1:**
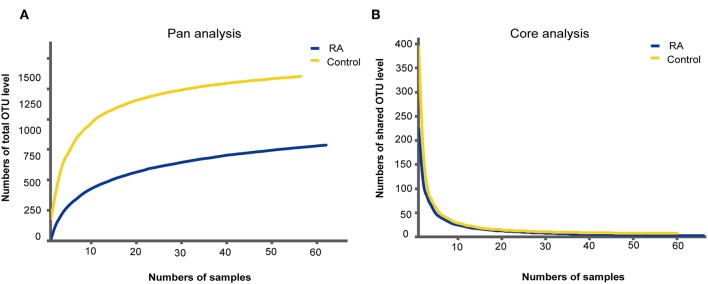
Accumulation curves for the pan **(A)** and core **(B)** species analysis of the RA group (blue lines) and control group (yellow lines).

A total of 126 samples from 66 RA patients and 60 healthy controls were analyzed in this study. The age and sex of all subjects are described in Table 1, and the sociodemographic details and pathological status of the RA patients are provided in Table 2. Detailed demographic and clinical characteristics of the study cohorts with RA are shown in Supplementary Table 1. Given a fact that RA occurs more frequently in women, female patients are more than male patients in this study. From these, 7,074,888 high-quality sequences with a median read length of 436 bp were acquired. Pan and core species analysis revealed increases in the number of species with increased sample sizes (Figure 1).

**Table 1:** Age and sex characteristics of study subjects.

**Group**	**Gender**	**Age**	**Number of subjects**
RA	Female	21–29	1
(*N =* 66)	(*N =* 51)	30–39	8
		40–49	9
		50–59	14
		60–69	16
		70–79	2
		>=80	1
	Male	50–59	6
	(*N =* 15)	60~69	9
Control	Female	20–29	5
(*N =* 60)	(*N =* 31)	30–39	2
		40–49	11
		50–59	10
		60–69	3
	Male	20–29	9
	(*N =* 29)	30–39	4
		40–49	6
		50–59	6
		>=60	4

**Table 2:** Sociodemographic characteristics and pathological status of the RA group.

**Variables**	**Values**
Male *n* (%)	15 (22.7%)
Age (years)	54.95 ± 1.4
Disease duration (years)	32 ± 0.6 (male patients)
	33.8 ± 0.2 (female patients)
Inflammatory markers	CR > 133 μmol/L (male patients)
	CR > 106 μmol/L (female patients)
	ESR > 15 mm/h (male patients)
	ESR > 20 mm/h (female patients)
	CRP > 10 mg/L
Serology	RF > 20
	Anti-CCP > 25
Joint involvement	15.9 ± 14.9 (male patients)
	24.5 ± 17.4 (female patients)

Values are shown as mean ± SD.

*SD, standard deviation; CR, Creatinine; ESR, Erythrocyte sedimentation rate; CRP, C-reactive protein; RF, Rheumatoid factor; CCP, Cyclic citrullinated peptide*.

### α-Diversity of the Gut Microbiome

**Figure 2:**
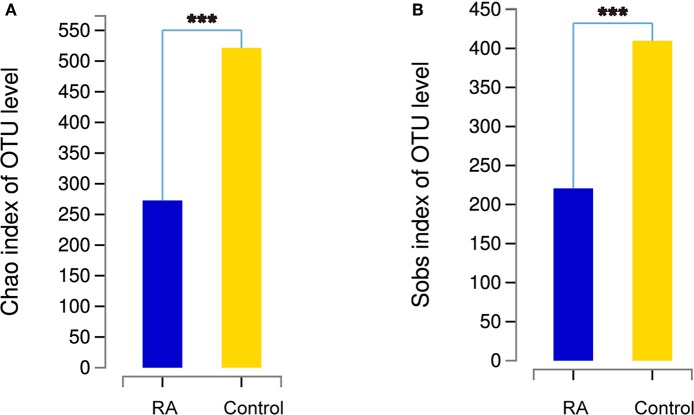
Comparison of the α-diversities of the gut microbiomes in the RA group and control group using the Chao index **(A)** and Sobs index **(B)**. Chao1 is an index that estimates the number of OTUs contained in a sample. It is commonly used in ecology to estimate the total number of species. Sobs is the observed number of OTUs. ^***^p < 0.001.

The α-diversity, determined using the Chao1 and Sobs indexes, differed significantly between the RA group and the healthy control group (Figure 2). The gut microbiome was markedly more diverse in composition in the control group than in the RA group. The data suggest a relationship between a decline in the α-diversity of the gut microbiome and the development of RA.

### β-Diversity of the Gut Microbiome

**Figure 3:**
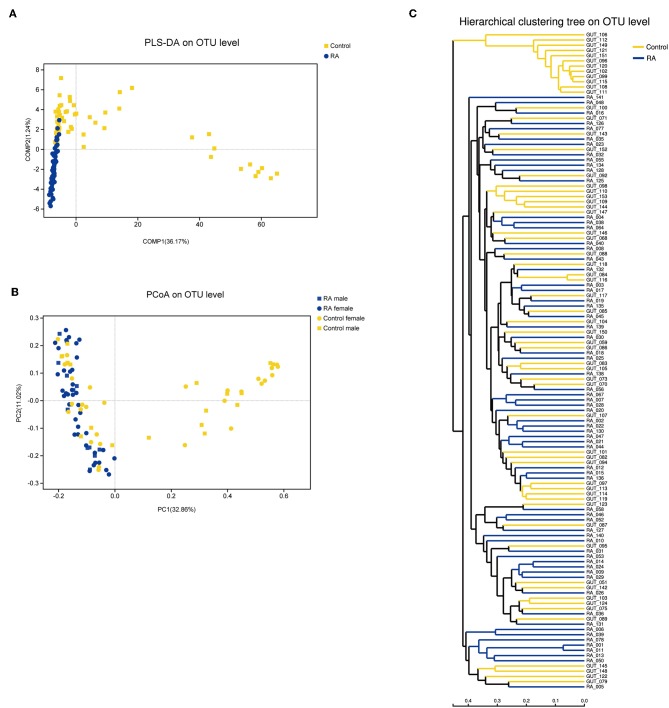
β-diversities of RA group and control group. **(A)** Partial least squares discriminant analysis (PLS-DA) of the RA and control groups. **(B)** Principal component analysis (PCoA) plot generated from the weighted UniFrac analysis. The x- and y-axes indicate the first and second coordinates, respectively, and the values in parentheses show the percentages of the community variation explained. The blue and yellow symbols depict microbial enrichment in the RA group and control group, respectively. **(C)** Hierarchical cluster analysis results obtained using UniFrac software.

Principal coordinate analysis based on operational taxonomic unit (OTU) abundances provided an overview of the gut microbiome and reflected the β-diversities of the different groups. The β-diversity was clearly higher in the control group than in the RA group (Figure 3). Among the control group, the bacterial communities in some fecal samples were similar to those of patients with RA, while the microbial composition of other individuals in the control group was completely distinct from that of the RA group. Samples from both males and females were included in this analysis and were found to differ in β-diversity.

### Microbiome Alterations

**Figure 4:**
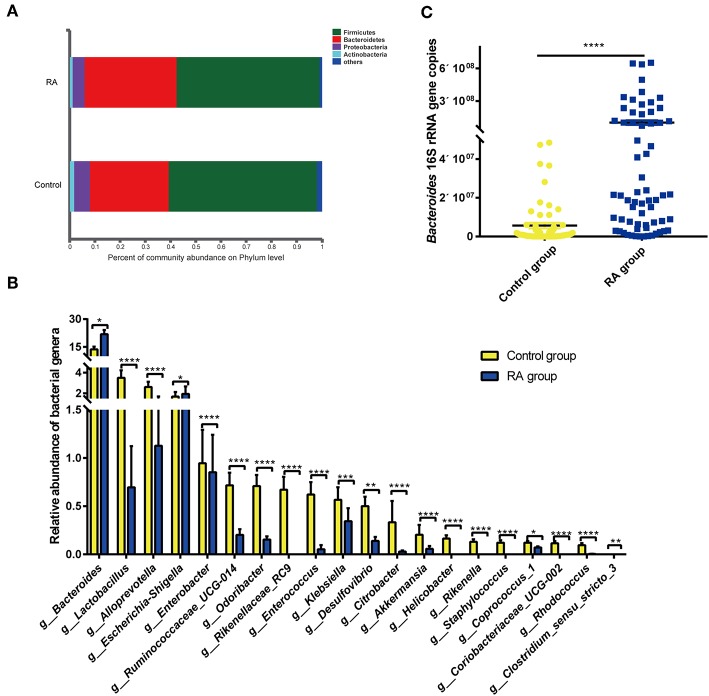
Differences in composition of the gut microbiome between the RA group and control group. **(A)** Prevalence of bacterial phyla. **(B)** Compositions of specific bacterial genera with the highest abundances in the RA and control cohorts. **(C)** qRT-PCR analysis of *Bacteroides* 16S rRNA genes. ^*^p < 0.05, ^**^p < 0.01, ^***^p < 0.001, ^****^p < 0.0001.

Bacteria in the phyla *Bacteroidetes, Firmicutes, Proteobacteria*, and *Actinobacteria* are the most enriched in the human intestine. The present data revealed the increased abundance of *Bacteroidetes* in RA patients, with decreased abundances of the other three bacterial phyla in the gut microbiome of RA patients (Figure 4A).

Of the total of 179 bacterial families, 126 were significantly different between the RA and control groups (*p* < 0.05; data not shown). Of the total of 438 bacterial genera, 221 differed markedly between the two groups (Supplementary Table 2). Significance tests were performed for the 20 most abundant bacterial genera that differed in abundance between the two groups, and the results are depicted in Figure 4B. RA group exhibited reductions in the genera *Lactobacillus, Alloprevotella, Enterobacter, Clostridium sensu stricto-3, Ruminococcaceae* UCG-014, *Odoribacter, Rikenellaceae* RC9, *Enterococcus, Klebsiella, Desulfovibrio, Citrobacter, Akkermansia, Helicobacter, Rikenella, Staphylococcus, Coprococcus* 1, *Coriobacteriaceae* UCG-002, and *Rhodococcus*. In contrast, nine bacterial genera, including *Bacteroides, Escherichia-Shigella, Parasutterella, Flavonifractor*, the *Eubacterium xylanophium* group, *Tyzzerella, Sellimonas*, and *Oscillospira*, were significantly increased in abundance in RA patients compared with that in healthy individuals (Supplementary Figure 1). The abundance of Bacteroides was confirmed by qPCR (Figure 4C). Circos plots were used to visualize the relationship between sample groups and specific bacterial families and genera (Supplementary Figure 2). Samples in the RA and control groups displayed similar bacterial members, although the abundances of many of these members differed substantially. *Lactobacillaceae* were evident in both groups, with the abundance being markedly higher in the control group.

### Bacterial Taxa Related to RA

To identify the specific bacterial taxa associated with RA, the compositions of the gut microbiomes of RA patients and healthy individuals were compared using the LDA effect size (LEfSe) method. The resulting cladogram presents the structure of the gut microbiome and the predominant bacteria in the control and RA groups. In the RA group, several bacterial genera, including *Bacteroides, Escherichia Shigella, Tyzzerella, Parasutterella, Flavonifractor, Eubacterium saphenum group*, and *Sellimonas*, could be considered microbial markers, as shown in Supplementary Figure 3.

### Functional Predictions Based on 16S rRNA Gene Sequencing

Inferred microbiome functions based on 16S rRNA gene sequences were also explored using the PICRUSt algorithm (Langille et al., 2013). Sixty Kyoto Encyclopedia of Genes and Genomes (KEGG) pathways, involving the biosynthesis of secondary metabolites, xenobiotics biodegradation and metabolism, metabolism of terpenoids and polyketides, immune system, and endocrine system, that differed in abundance between the RA group and the control group at *q* ≤ 0.05 are shown in the heatmap in Supplementary Figure 4. The implicated pathways did not obviously differ among the different bacterial phyla. Most pathways in the heatmap were enriched in the healthy controls but depleted in the RA group. Notably, the genus *Bacteroides* was depleted in most of these pathways. Interestingly, the pathway related to steroid hormone biosynthesis, which includes sex hormones, was enriched in *Bacteroides* but depleted in other bacterial genera.

### Correlation Between Microbiota and Physiological Measures of RA

**Figure 5:**
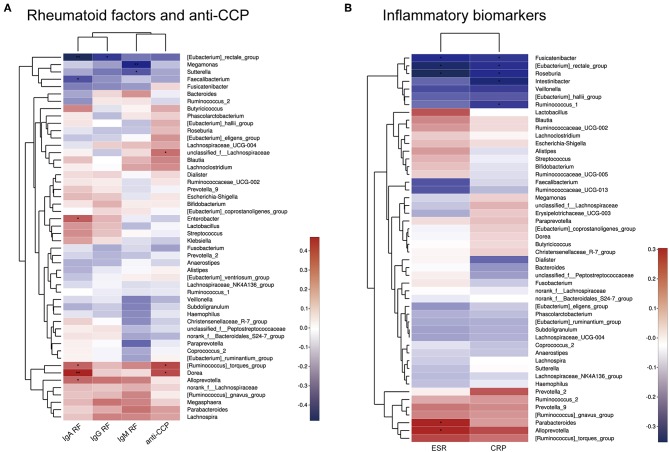
Heatmap of Spearman correlations between bacterial taxa and rheumatoid factors and anti-CCP antibodies **(A)** or inflammatory biomarkers **(B)**. ^*^p < 0.05, ^**^p < 0.01.

Spearman correlation analysis of rheumatoid factors (RFs), anti-CCP antibodies, inflammatory biomarkers, and the most abundant 50 bacterial genera among the intestinal microbiota was performed. As shown in Figure 5, positive correlations (correlation coefficient > 0) and negative correlations (correlation coefficient < 0) were observed between various bacteria and rheumatoid factors, anti-CCP antibodies, and inflammatory biomarkers.

## Discussion

The differences in the gut microbiome between RA patients and healthy individuals have been demonstrated systematically since the beginning of this century. Nevertheless, details of the microbiome of RA patients in the Chinese population remained unclear. In the current study, we recruited a large cohort of 126 individuals to study the characteristics of the gut microbiome composition in RA patients in China.

The gut microbiome of these RA patients showed a decline in species richness as well as a moderate reduction in β-diversity, consistent with previous reports (Chen et al., 2016; Van de Wiele et al., 2016). The compositional characteristics of the gut microbiome in the RA group were like the overall characteristics of most samples in the control group but were distinct from some samples in the control group. This collection of control individuals contained both males and females with no clear defining characteristic, indicating that the composition of the gut microbiome differed markedly among those in the healthy cohort but not among patients with RA. The results indicate that many individuals with specific compositional characteristics of the gut microbiome are more susceptible to RA, which may aid in the diagnosis or determination of the susceptibility of individuals to RA via detection of the gut microbiome.

At the genus level, *Lactobacillus* was depleted in the RA group compared to its abundance in the control group. *Lactobacilli* are typical probiotic microorganisms found in the normal bacterial flora. They exhibit immunoregulatory functions in the host and play important roles in the maintenance of homeostasis in the intestinal microbiota by secreting immunomodulatory agents (Lorca et al., 2001; Zvanych et al., 2014). However, some species of *Lactobacillus*, such as *L. bifidus*, can cause joint swelling in mice (Abdollahi-Roodsaz et al., 2008). Several studies have reported a greater abundance of *Lactobacillus* in RA patients than in healthy controls in the gut microbiome and the oral and salivary microbiome (Liu et al., 2013; Van de Wiele et al., 2016). Conversely, in this study, the abundance of *Lactobacillus* was significantly reduced in RA patients compared to that in the control group. Many studies, including several randomized, double-blinded, placebo-controlled trials, have demonstrated that supplementation with *L*.GG, *L. casei*, or *Lactobacillus*-enriched vegetarian food can moderately improve health and attenuate inflammation in RA patients (Nenonen et al., 1998; Hatakka et al., 2003; de los Angeles Pineda et al., 2011; Alipour et al., 2014).

At the phylum level, RA patients displayed an increase in *Bacteroidetes* and decreases in *Firmicutes, Proteobacteria*, and *Actinobacteria* compared to levels in healthy individuals. Differences in the abundances of the genus *Bacteroides* between the RA group and control group accounted for the majority of differences within the phylum *Bacteroidetes*. *Bacteroides* species are reported to mediate chronic colitis, gastritis, and arthritis in HLA-B27 transgenic rats (Rath et al., 1996) and to cause osteomyelitis in RA (Crook and Gray, 1982). Studies in germ-free mice have demonstrated that the *Bacteroides* integrase antigen contributes to the recruitment and proliferation of low-avidity CD8^+^ T cells, which may be analogous to thymic CD4^+^ Tregs, and the response to chronic antigenic exposure in gut-associated lymphoid tissues (Hebbandi Nanjundappa et al., 2017). The enrichment of *Bacteroidetes* in RA patients in this study indicates that this condition may contribute to disease progression. Additionally, the expansion of *Bacteroidetes* may be attributed to a compensatory mechanism for downregulating autoimmune reactions.

The microbiota, especially certain microbes, can influence human physiology and pathology. Functional analysis of the KEGG pathways related to 16S rRNA gene sequences and differences in the microbiomes of RA patients and healthy controls provided evidence that some microbe-derived functions, including the biosynthesis of some secondary metabolites, degradation of xenobiotics, metabolism, and the endocrine system, may be depleted in patients with RA. However, these pathways were also depleted in *Bacteroides*, a bacterial genus enriched in the RA group. These findings indicate that the composition of the gut microbiota may contribute to the initiation or development of RA through altered microbial function. *Bacteroides* might play a crucial role in this process.

Moreover, there were significant relationships between physiological measures of RA and the microbiota, as shown by Spearman correlation analysis. For example, *Sutterella, Megamonas*, and *Faecalibacterium* were negatively correlated with RF levels. Other bacteria, such as *Dorea* and *Alloprevotella*, were positively correlated with the RF levels or anti-CCP antibodies. This indicates that specific bacteria may contribute to symptoms of RA. However, it is difficult to determine the exact influence of the bacterial population on RA, as isolation and *in vitro* culture of most microbial strains is challenging.

The development of RA is associated with gender. The morbidity of RA is higher in women than in men (van Vollenhoven, 2009). Previous studies demonstrated that sex hormones can induce autoimmune diseases through an effect on the function of the immune system (Ahmed et al., 1985). Many microbiota functions predicted using bacterial 16S rRNA gene sequencing involved the endocrine system and steroid hormones, which may play a role in RA. An experiment in mice showed that interferon-gamma is the predominant inflammatory cytokine after the activation of T cells in female mice, indicating that the Th1 response is more intense in female mice, while the Th2 response is dominant in male mice (Bebo et al., 1999). Female hormones can regulate the activity of T cells, B cells, natural killer cells, macrophages, endotheliocytes, and matrix cells. Moreover, T cells are the main target cells of female hormones. Thus, levels of female hormones can result in the dysbiosis of Tregs. However, women are not more susceptible to some other autoimmune diseases, including ankylosing spondylitis. A study of a non-obese mouse model of type 1 diabetes revealed that the sex-related microbiome can drive hormone-dependent regulation of autoimmunity (Calin et al., 2000). In the present study, LEfSe analysis of the gut microbiome compositions of males and females showed that *Rikenellaceae, Porphyromonadaceae, Coriobacteriaceae*, and certain genera were significantly more abundant in females, while *Pasteurellaceae, Butyricicoccus, Clostridiaceae 1, Clostridium sensu stricto 1, Alisnonella*, and other genera were more abundant in males according to linear discriminant analysis (LDA) scores (Supplementary Figure 5). The members of the *Clostridiaceae* enriched in males are reported to stimulate the differentiation of colonic regulatory T cells (Hasegawa and Inohara, 2014). However, we did not conclusive*ly* demonstrate that differences in the composition of the gut microbiome between males and females influence the development of RA in this study, which was limited by the sample size to some extent. Except for gender, given the complexity of clinical samples collection, we cannot rule out the influence of age on experimental results, although the results in this study is solid. We performed statistics on the ages of the RA group and the control group and found that the former was significantly higher than the latter (data not shown). Despite this, such age groups are closer to the real situation in clinical cases. Therefore, we believe that age and gender are the co-influence factors of the bacterial population of RA patients, but our results still have considerable guiding significance.

Studies of the gut microbiome in Chinese patients with RA can contribute to the recognition of the microbiome composition in patients with autoimmune diseases. Such altered bacterial collections can act as an adjuvant criterion for clinical diagnosis. Further research using different enterotypes and genders is needed, since the normal gut microbiome may exhibit marked differences in the enrichment of some bacteria, such as *Bacteroides* spp., *Prevotella* spp., and *Ruminococcus* spp. (Jeffery et al., 2012).

The contribution of the fungiome and virome to human health or disease has been highlighted recently. A study based on real-time PCR detected cytomegalovirus, Epstein–Barr virus, and human herpesvirus 6 in the sera and peripheral blood mononuclear cells of patients with RA (Alvarez-Lafuente et al., 2005). Intestinal fungiome and virome analyses are theoretically included in analyses of the microbiome, although the actual results may be limited by technological complexities. Further studies to reveal the detailed bacteriome, virome, and fungiome characteristics in RA patients with different enterotypes are therefore warranted.

## Conclusions

In this study, we showed that the diversity and composition of the microbiome of RA patients differed from those in healthy control subjects in China. An increase in *Bacteroides* and *Escherichia*-*Shigella*, combined with decreases in *Lactobacillus, Alloprevotella, Enterobacter*, and *Odoribacter* appear to be characteristic of RA in patients from Shanghai, China. These findings differ from those of other studies performed outside of China. Spearman correlation analysis showed that *Dorea* and *Ruminococcus* were positively correlated with RF-IgA and anti-CCP antibodies. Similarly, *Alloprevotella* was positively correlated with rheumatoid factors (RF-IgM, RF-IgA, and RF-IgG) and inflammation biomarkers (CRP, ESR). The roles of these bacteria in the etiology and pathogenesis of RA require further exploration, as this analysis was limited by the small sample size. Future studies should also evaluate the significance of these microbial changes for the diagnosis, prognosis, and drug response of RA.

## Data Availability Statement

The datasets generated for this study can be found in NCI SRA https://www.ncbi.nlm.nih.gov/sra/SRP223483.

## Ethics Statement

This study was approved by the Institutional Review Board (IRB) at Shanghai GuangHua Hospital of Integrated Traditional Chinese and Western Medicine. All participants gave written informed consent in line with the Declaration of Helsinki.

## Author Contributions

LX, Y-WT, ZP, HL, and XG designed the study. YS, QC, and PL conducted analyses and wrote the manuscript. YS, QC, LY, QL, and CL edited the manuscript. RX, DH, WJ, YB, YS, and KD provided sociodemographic and pathological data.

### Conflict of Interest

The authors declare that the research was conducted in the absence of any commercial or financial relationships that could be construed as a potential conflict of interest.
