# Supplementary material for: Characteristics of Gut Microbiota in Patients With Rheumatoid Arthritis in Shanghai, China
Source: Front Cell Infect Microbiol. 2019 Oct 23;9:369. doi: 10.3389/fcimb.2019.00369 (PMC6819506; doi:10.3389/fcimb.2019.00369)
Supplement: Supplementary file 1 [file Data_Sheet_1.pdf]

# *Supplementary Material*

## 1 Supplementary Figures and Tables

### 1.1 Supplementary Figures

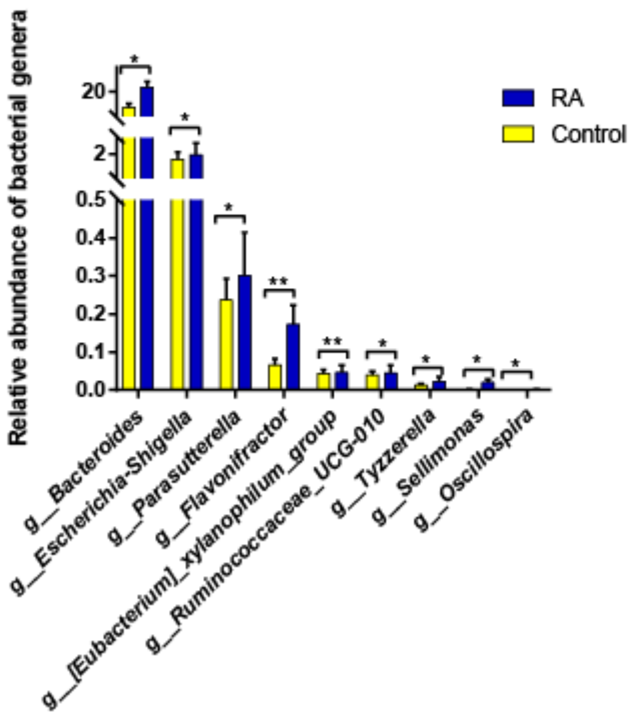


**Supplementary Fig. 1** Bacterial genera with significant higher abundance in the RA group.

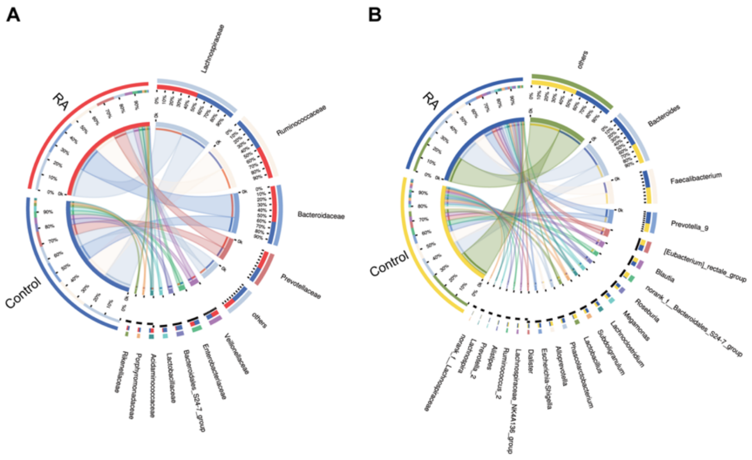


**Supplementary Fig. 2** Differences in composition of the gut microbiome between the RA group and control group. (A)Circos plots were used to visualize the relationship between sample groups and specific bacterial families and (B)genera .

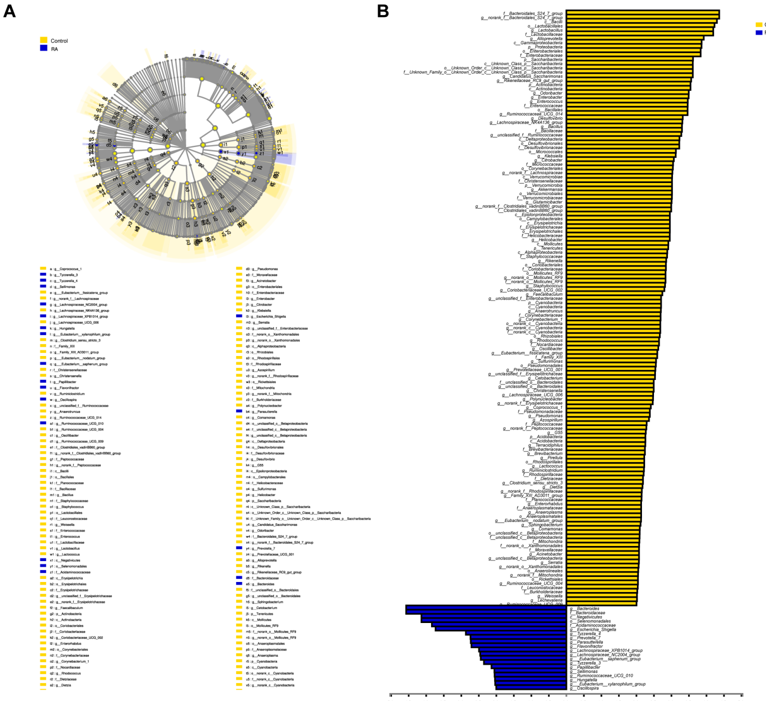


**Supplementary Fig. 3** Differences in abundances of bacterial communities between the RA and healthy control groups. (A) Taxonomic cladogram obtained by LEfSe analysis revealing differences in abundances of bacterial communities. RA-enriched and healthy control-enriched taxa are depicted in blue and yellow, respectively. (B) The LDA scores of the RA group and the healthy control group. Histograms in blue and yellow indicate the enriched bacterial taxa in RA patients and healthy control individuals, respectively.

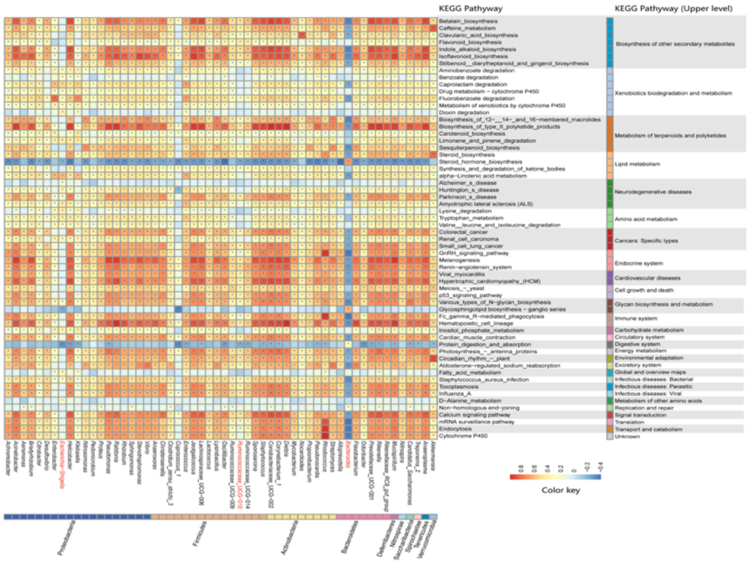


**Supplementary Fig. 4** Bacterial taxa associated with RA related to gene functional pathways. Bacterial gene functions were predicted from 16S rRNA gene-based microbial compositions using the PICRUSt algorithm to make inferences from the KEGG annotated databases (Wu et al. 2016). Spearman's correlation coefficients were estimated for each pairwise comparison of genus and KEGG pathway counts. All KEGG pathways exhibiting a significant difference between the RA and control groups are included, and bacterial genera of interest are shown in the heatmap. The bacterial genera in red were enriched, while the remainder were depleted in the RA group.

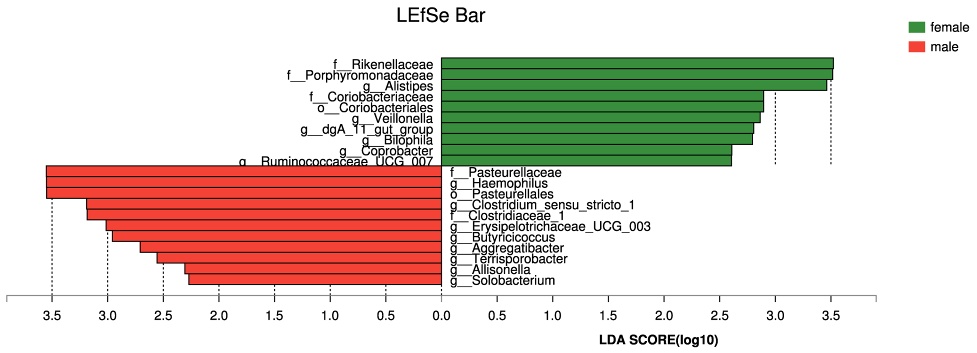


**Supplementary Fig. 5** Differences in abundances of bacterial communities between males and females. Linear discriminant analysis (LDA) score of males and females  Histograms in green show enriched bacterial taxa in females, and those in red show enriched bacterial taxa in males.

## 1.2 Supplementary Tables

Supplementary Table 1. Demographic and clinical characteristics of the study cohort with RA

| | | | | | Blood routine examination | | | | Routine urine examination | | | | | | |
|---|---|---|---|---|---|---|---|---|---|---|---|---|---|---|---|
| Sample ID | Gender | Age | Tender joint counts | Swell joint counts | Leukocyte ($\times 10^9$/L) | Hemoglobin ($\times$g/L) | Erythrocyte ($\times 10^{12}$/L) | Thrombocyte ($\times 10^9$/L) | Urine protein (g/L) | Urine specific gravity | PH | ketone body | Urine UGLU | Urine RBC (/ul) | LEU (/ul) |
| RA -001 | F | 60 | 4 | 4 | 6.93 | 126 | 4.15 | 242 | - | 1.005 | 8 | - | normal | 0-1/HP | 2-3/HP |
| RA -002 | F | 35 | 21 | 11 | 7.79 | 140 | 4.4 | 194 | - | 1.005 | 8 | - | normal | 0-1/HP | 2-3/HP |
| RA -003 | M | 65 | 7 | 6 | 5.39 | 129 | 4.17 | 242 | - | 1.02 | 6 | - | normal | 0-1/HP | 2-3/HP |
| RA -004 | F | 44 | 8 | 4 | 5.86 | 100 | 3.71 | 266 | - | 1.015 | 8 | - | normal | 0-1/HP | 2-3/HP |
| RA -005 | M | 61 | 28 | 3 | 9.61 | 150 | 4.59 | 325 | | | | | | | |
| RA -006 | F | 61 | 22 | 22 | 5.63 | 133 | 4.1 | 197 | - | 1.025 | 6 | - | normal | 0-1/HP | 2-3/HP |
| RA -007 | F | 49 | 3 | 4 | 7.06 | 122 | 4.44 | 227 | - | 1.01 | 7 | - | normal | 0-1/HP | 2-3/HP |
| RA -008 | F | 37 | 2 | 2 | 9.6 | 145 | 4.64 | 282 | - | 1.015 | 5 | + | normal | 0-1/HP | 2-3/HP |
| RA -009 | F | 27 | 6 | 1 | 9.12 | 121 | 4.57 | 286 | - | 1.01 | 8 | - | normal | 0-1/HP | 2-3/HP |
| RA -010 | F | 56 | 4 | 0 | 3.3 | 85 | 2.6 | 154 | - | 1.03 | 5 | - | normal | 0-1/HP | 4-5/HP |
| RA -011 | F | 49 | 6 | 0 | 9.68 | 126 | 4.22 | 241 | - | 1.025 | 5 | - | + | 0-1/HP | 2-3/HP |
| RA -012 | F | 42 | 18 | 2 | 7.56 | 128 | 4.63 | 290 | - | 1.02 | 6 | - | normal | 0-1/HP | 2-3/HP |
| RA -013 | F | 54 | 13 | 1 | 4.62 | 109 | 3.7 | 216 | - | 1.02 | 5 | - | normal | 0-1/HP | 2-3/HP |
| RA -014 | M | 63 | 1 | 2 | | | | | | | | | | | |
| RA -015 | M | 54 | 18 | 9 | 9.57 | 155 | 5.09 | 168 | - | 1.02 | 6 | - | normal | 0-1/HP | 2-3/HP |
| RA -016 | F | 62 | 1 | 1 | | | | | | | | | | | |
| RA -017 | M | 63 | 26 | 18 | 8.78 | 148 | 4.73 | 191 | - | 1.02 | 5 | - | + | 0-1/HP | 2-3/HP |
| RA -018 | F | 61 | 4 | 2 | 6.91 | 130 | 4.15 | 205 | - | 1.025 | 7 | - | normal | 0-1/HP | 2-3/HP |
| RA -019 | F | 34 | 14 | 12 | 7.35 | 140 | 4.41 | 265 | - | 1.015 | 6 | - | normal | 0-1/HP | 2-3/HP |
| RA -020 | F | 73 | 3 | 1 | | | | | | | | | | | |
| RA -021 | F | 51 | 11 | 0 | 4.53 | 143 | 4.57 | 197 | - | 1.015 | 7 | - | normal | 0-1/HP | 2-3/HP |
| RA -022 | F | 35 | 20 | 20 | 3.6 | 121 | 4.13 | 159 | - | 1.015 | 6 | - | normal | 0-1/HP | 2-3/HP |
| RA -023 | F | 52 | 15 | 0 | 8.26 | 110 | 4.2 | 360 | - | 1.02 | 7 | - | normal | 0-1/HP | 2-3/HP |

| | | | | | | | | | | | | | | | |
|---|---|---|---|---|---|---|---|---|---|---|---|---|---|---|---|
| RA -024 | F | 69 | 4 | 2 | 6.3 | 152 | 5 | 243 | - | 1.025 | 5 | - | normal | + | 2-3/HP |
| RA -025 | M | 64 | 6 | 2 | 8.32 | 117 | 3.94 | 433 | - | 1.025 | 5 | - | normal | 0-1/HP | 2-3/HP |
| RA -026 | M | 54 | 18 | 8 | | | | | - | 1.025 | 5 | - | normal | +++ | 2-3/HP |
| RA -028 | F | 58 | 1 | 0 | 4.84 | 145 | 4.67 | 255 | - | 1.015 | 6 | - | normal | +++ | 7-8/HP |
| RA -029 | M | 62 | 28 | 18 | 7.06 | 111 | 3.44 | 142 | - | 1.03 | 5 | - | normal | 0-1/HP | 2-3/HP |
| RA -030 | F | 58 | 22 | 1 | 9.43 | 128 | 4.15 | 204 | - | 1.02 | 5 | - | normal | 0-1/HP | 2-3/HP |
| RA -031 | F | 54 | 2 | 2 | 6.13 | 134 | 5.12 | 195 | - | 1.01 | 6 | - | normal | 0-1/HP | 2-3/HP |
| RA -032 | F | 64 | 4 | 1 | 6.7 | 128 | 4.11 | 175 | - | 1.015 | 5 | - | normal | 0-1/HP | 2-3/HP |
| RA -035 | F | 55 | 13 | 0 | 4.43 | 144 | 4.9 | 189 | - | 1.01 | 8 | - | normal | 0-1/HP | 3-4/HP |
| RA -036 | F | 51 | 0 | 0 | | | | | | | | | | | |
| RA -038 | F | 40 | 7 | 4 | 4.98 | 91 | 3.85 | 386 | - | 1.02 | 5 | - | normal | 0-1/HP | 3-5/HP |
| RA -039 | F | 62 | 5 | 0 | 7.14 | 123 | 4.01 | 258 | - | 1.025 | 7 | - | normal | 1-3/HP | 3-4/HP |
| RA -040 | M | 51 | 3 | 0 | 8.8 | 167 | 5.44 | 195 | - | 1.02 | 6 | - | normal | 0-1/HP | 2-3/HP |
| RA -043 | M | 68 | 23 | 18 | 8.02 | 141 | 4.64 | 171 | - | 1.025 | 6 | - | normal | 0-1/HP | 2-3/HP |
| RA -044 | M | 51 | 26 | 18 | 5.5 | 139 | 4.91 | 221 | - | 1.025 | 6 | - | normal | 0-1/HP | 2-3/HP |
| RA -045 | F | 52 | 24 | 10 | 6.1 | 105 | 4.34 | 260 | - | 1.015 | 7 | - | normal | 0-1/HP | 2-3/HP |
| RA -046 | F | 58 | 4 | 0 | 4.76 | 122 | 4.09 | 215 | - | 1.015 | 7 | - | normal | 0-1/HP | 2-3/HP |
| RA -047 | F | 40 | 10 | 0 | 6.54 | 121 | 3.77 | 327 | - | 1.005 | 7 | - | normal | 0-1/HP | 2-3/HP |
| RA -048 | F | 68 | 48 | 0 | | | | | | | | | | | |
| RA -050 | F | 65 | 9 | 8 | 6.22 | 114 | 4.02 | 276 | - | 1.02 | 5 | - | normal | 0-1/HP | 2-3/HP |
| RA -052 | F | 68 | 9 | 3 | | | | | | | | | | | |
| RA -053 | F | 46 | 7 | 20 | 5.73 | 137 | 5.41 | 242 | | | | | | | |
| RA -055 | F | 65 | 14 | 12 | 6.24 | 120 | 3.85 | 126 | | | | | | | |
| RA -056 | F | 72 | 27 | 15 | 9.83 | 102 | 3.33 | 384 | - | | | - | normal | "+++ | 2-3/HP |
| RA -058 | F | 58 | 22 | 20 | 4.18 | 109 | 3.71 | 299 | - | 1.015 | 6 | - | normal | 0-1/HP | 2-3/HP |
| RA -064 | F | 63 | 12 | 4 | | | | | | | | | | | |
| RA -067 | M | 67 | 9 | 12 | 8.12 | 120 | 4.45 | 319 | | | | | | | |
| RA -077 | F | 33 | 1 | 1 | 6.92 | 117 | 4.04 | 305 | - | 1.025 | 5 | - | normal | 0-1/HP | 3-4/HP |
| RA -078 | F | 64 | 25 | 20 | 4.68 | 123 | 4.54 | 169 | - | | | | | | |

| | | | | | | | | | | | | | | | |
|---|---|---|---|---|---|---|---|---|---|---|---|---|---|---|---|
| RA -125 | M | 55 | | | | | | | | | | | | | |
| RA -126 | F | 68 | | | 5.3 | 129 | 4 | 146 | | | | | | | |
| RA -127 | F | 58 | | | | | | | | | | | | | |
| RA -128 | F | 47 | | | | | | | | | | | | | |
| RA -130 | F | 67 | 5 | 3 | 5.87 | 141 | 4.64 | 236 | - | 1.005 | 6 | - | normal | 0-1/HP | 2-3/HP |
| RA -131 | F | 52 | 20 | 0 | 5.19 | 110 | 6.11 | 122 | | | | | | | |
| RA -132 | M | 62 | 1 | 1 | 5.84 | 118 | 3.82 | 179 | - | 1.025 | 5 | - | normal | 6-7/HP | 4-6/HP |
| RA -134 | F | 81 | 10 | 2 | 6.45 | 109 | 3.42 | 281 | | | | | | | |
| RA -135 | F | 40 | 10 | 6 | 7.69 | 117 | 4.13 | 206 | - | 1.025 | 7 | - | normal | 0-1/HP | 2-3/HP |
| RA -136 | F | 39 | 8 | 8 | 5.63 | 130 | 4.39 | 172 | - | 1.015 | 7 | - | normal | 0-1/HP | 2-3/HP |
| RA -138 | F | 62 | 26 | 14 | | | | | - | 1.005 | 6 | - | normal | | |
| RA -139 | F | 38 | 36 | 0 | 11.8 | 115 | 3.85 | 304 | | | | | | | |
| RA -140 | M | 53 | 11 | 15 | 10.34 | 157 | 5.26 | 238 | - | 1.025 | 5 | - | normal | 0-1/HP | 2-3/HP |
| RA -141 | F | 37 | 13 | 8 | 6.51 | 118 | 4.74 | 328 | - | 1.03 | 5 | - | normal | 0-1/HP | 2-3/HP |

Continued

| Sample ID | Blood biochemistry | | | | | | Inflammatory markers | | Serological indicator | | | |
|---|---|---|---|---|---|---|---|---|---|---|---|---|
| | ALT (U/L) | AST (U/L) | ALP (U/L) | GGT (U/L) | BUN (U/L) | Cr (U/L) | ESR (mm/h) | CRP (mg/L) | RF（lu/ml） | | | ACCP (U/ml) |
| | | | | | | | | | RF-IgM | RF-IgA | RF-IgG | |
| RA -001 | 10 | 15 | | | | 41 | 33 | 22.96 | <20 | 0.4 | 2.15 | <25 |
| RA -002 | 13 | 18 | | | | | 4 | 0.44 | <20 | 0.1 | 0.2 | <25 |
| RA -003 | 11 | 17 | | | | 53 | 27 | 6.87 | 130 | 1.08 | 3.86 | 1600 |
| RA -004 | 9 | 18 | 59 | 10 | 3 | 34 | 119 | 43.22 | 2300 | | | |
| RA -005 | 18 | 18 | | | | 52 | 14 | 15.63 | 20.4 | >300 | 53.05 | 1600 |
| RA -006 | 15 | 24 | | | | 52 | 23 | 1.2 | 126 | 9.3 | 13.1 | 283.52 |
| RA -007 | 13 | 20 | | | | 41 | 38 | 0.8 | 67.4 | 36.56 | 59.76 | 1098.2 |
| RA -008 | 9 | 12 | | | | 48 | 2 | 2.96 | <20 | 3.85 | 10.36 | <25 |
| RA -009 | 15 | 19 | | | | 47 | 53 | 16.63 | 27.1 | 5.97 | 21.51 | 314.51 |
| RA -010 | 10 | 22 | | | 4.8 | 49 | 109 | 6.6 | 653 | 25.52 | 83.9 | <25 |
| RA -011 | 16 | 12 | | | | | 21 | 0.69 | 20.6 | 3.64 | 36.03 | <25 |
| RA -012 | 11 | 17 | | | 7 | 53 | 7 | 3.22 | <20 | 0.13 | 1.82 | <25 |
| RA -013 | 11 | 14 | 51 | 17 | 7.3 | 72 | 53 | 12.39 | 390 | 149.57 | 20.8 | 967.52 |
| RA -014 | | | | | | | | | | | | |
| RA -015 | 9 | 14 | | | | 55 | 16 | 9.16 | 123 | 17.1 | 99.97 | <25 |
| RA -016 | | | | | | | | | | | | |

| | | | | | | | | | | | | |
|---|---|---|---|---|---|---|---|---|---|---|---|---|
| RA -017 | 17 | 20 | 54 | 27 | 7.6 | 72 | 19 | 6.02 | 621 | 37.61 | 139.11 | 1600 |
| RA -018 | 37 | 33 | 69 | 30 | 5.6 | 48 | 22 | 2.75 | 81.3 | 0.48 | 3.55 | <25 |
| RA -019 | 14 | 18 | 80 | 16 | | 47 | 14 | 1.64 | <20 | 0.31 | 1.5 | 164.55 |
| RA -020 | | | | | | | | | | | | |
| RA -021 | 17 | 27 | | | | | 8 | 0.54 | <20 | 1.28 | 0.99 | <25 |
| RA -022 | 18 | 20 | | | | 30 | 23 | 0.5 | 69.9 | 9.85 | 34.36 | 85.46 |
| RA -023 | 5 | 16 | 88 | 16 | 5.3 | 35 | 77 | 13.44 | 547 | 104.65 | 90.88 | 77.07 |
| RA -024 | 22 | 28 | | | 4.2 | 49 | 10 | 0.96 | <20 | 0.85 | 2.55 | <25 |
| RA -025 | | | | | | | 68 | 24.19 | 22.4 | 5.33 | 4.86 | 195.39 |
| RA -026 | | | | | | | | | 23.1 | 38.46 | >300 | 107.88 |
| RA -028 | 32 | 23 | | | 42 | 60 | 15 | 0.92 | <20 | 3.09 | 1.59 | <25 |
| RA -029 | 50 | 42 | | | 10.9 | 55 | 104 | 83.01 | 148 | 11.81 | 66.49 | 45.58 |
| RA -030 | 10 | 17 | | | | 41 | 30 | 2.04 | 491 | 15.36 | 95.37 | 305.72 |
| RA -031 | 6 | 17 | | | 3.4 | 55 | 26 | 5.77 | 242 | 211.54 | 85.99 | 192.75 |
| RA -032 | 11 | 15 | | | 6.9 | 238 | 31 | 0.5 | 209 | 57.82 | 97.86 | 343.25 |
| RA -035 | | | | | | | 10 | 0.5 | | | | |
| RA -036 | | | | | | | | | | | | |
| RA -038 | 7 | 9 | 74 | 14 | 4.8 | 42 | 110 | 78.4 | 411 | 9.85 | 94.6 | |
| RA -039 | 10 | 16 | | | 4.2 | 48 | 30 | 0.4 | 258 | | | 1600 |
| RA -040 | 14 | 18 | 60 | 22 | 7 | 55 | 4 | 2.64 | <20 | 58.09 | 29.42 | 67.57 |
| RA -043 | 12 | 17 | 86 | 14 | 6 | 72 | 16 | 2.45 | 263 | >300 | 33.66 | |
| RA -044 | 9 | 15 | 82 | 14 | 8.7 | 65 | 18 | 0.5 | <20 | 2.24 | 0.6 | <25 |
| RA -045 | 10 | 12 | 63 | 12 | 3.3 | 40 | 37 | 4.77 | 209 | 1.86 | 15.14 | 1130.91 |
| RA -046 | 39 | 33 | | | | 50 | 9 | 0.33 | <20 | 0.15 | 0.29 | 44.23 |
| RA -047 | 12 | 20 | 60 | 10 | 3.4 | 54 | 48 | 7.9 | 75.9 | 5.02 | 30.49 | 625.64 |
| RA -048 | | | | | | | | | | | | |
| RA -050 | 8 | 15 | 98 | 15 | 5.2 | 70 | 80 | 26.86 | | | | |
| RA -052 | | | | | | | | | | | | |
| RA -053 | 36 | 42 | 118 | 49 | 3.4 | 49 | 36 | 0.5 | 21.4 | 42.03 | 20.85 | 147.06 |

| | | | | | | | | | | | | |
|---|---|---|---|---|---|---|---|---|---|---|---|---|
| RA -055 | | | | | 4.5 | 52.4 | 26 | 17 | 162.2 | | | |
| RA -056 | 10 | 10 | 65 | 24 | 7.8 | 51 | 75 | 13.31 | >2460 | 169.94 | 230.68 | 73.12 |
| RA -058 | | | | | | | 70 | 62.01 | <20 | | | <25 |
| RA -064 | | | | | | | | | | | | |
| RA -067 | 10 | 16 | 97 | 16 | 8.5 | 61 | 116 | 81.47 | 10 | | 49 | 738 |
| RA -077 | 6 | 13 | | | 268 | 52 | 36 | 4.87 | 304 | >300 | 79.26 | 1213 |
| RA -078 | 63 | 42 | | | | 52 | 27 | | 478 | 10.14 | 119.07 | <25 |
| RA -125 | | | | | | | | | | | | |
| RA -126 | | | | | | <5 | 2 | | | | | <25 |
| RA -127 | | | | | | | | | | | | |
| RA -128 | | | | | | | | | | | | |
| RA -130 | 22 | 28 | | | 40.2 | 0.5 | 14 | 0.7 | 463 | 6 | 63 | 1071 |
| RA -131 | 19 | 18 | | | 47.5 | | 7 | 0.85 | <20 | 3.8 | 1.3 | <25 |
| RA -132 | 44 | 30 | | | | 61.2 | 25 | 0.6 | 572 | 1.3 | 58 | <25 |
| RA -134 | | | | | | | 70 | 38.06 | <20 | 1.3 | 4.2 | <25 |
| RA -135 | | | | | | | 37 | 2.3 | 114 | 66.94 | 79.45 | 170.57 |
| RA -136 | 11 | 16 | | | | | 12 | 0.91 | <20 | 1.4 | 4.1 | <25 |
| RA -138 | | | | | | | | | | | | |
| RA -139 | 24 | 23 | | | 4.26 | 46 | 97 | 111.02 | | | | |
| RA -140 | 17 | 18 | 114 | | 4.2 | 51.2 | 5 | 1.84 | 36.2 | 69 | 8 | 323 |
| RA -141 | 13 | 16 | 59 | 17 | 3.7 | 42.5 | 19 | 0.96 | 283 | 109 | 133 | 1117 |

ALT = alanine aminotransferase, AST= aspartate amino transferase, ALP = alkaline phosphatase, GGT = gamma-glutamyl transpeptidase, BUN = blood urea nitrogen, Cr = creatinine, ESR = erythrocyte sedimentation rate, CRP = C-reactive protein

Supplementary Table 2. Bacterial genera significantly altered between RA group and healthy control (GUT) group

| Species name | GUT-Mean (%) | GUT-SD (%) | RA-Mean (%) | RA-SD (%) | P-value | Corrected p-value | Lower CI | Upper CI | Effect size |
|---|---|---|---|---|---|---|---|---|---|
| *g__Rikenellaceae_RC9_gut_group* | 0.6708 | 1.1 | 9.46E-05 | 0.00054 | 4.55E-14 | 1.02E-11 | -0.9557 | -0.3886 | -0.6708 |
| *g__Helicobacter* | 0.1642 | 0.2777 | 0 | 0 | 4.67E-14 | 1.02E-11 | -0.2385 | -0.0962 | -0.1642 |
| *g__Rhodococcus* | 0.09505 | 0.1819 | 0.004259 | 0.01258 | 2.31E-13 | 3.38E-11 | -0.1459 | -0.05103 | -0.09079 |
| *g__Candidatus_Saccharimonas* | 0.7426 | 1.303 | 0.000379 | 0.001287 | 1.88E-12 | 2.06E-10 | -1.073 | -0.4138 | -0.7422 |
| *g__Coriobacteriaceae_UCG-002* | 0.1147 | 0.1953 | 0 | 0 | 2.38E-12 | 2.09E-10 | -0.1648 | -0.0685 | -0.1147 |
| *g__Glutamicibacter* | 0.186 | 0.3246 | 9.46E-05 | 0.00054 | 7.38E-12 | 4.04E-10 | -0.2707 | -0.1119 | -0.1859 |
| *g__Faecalibaculum* | 0.1014 | 0.1676 | 4.73E-05 | 0.000384 | 7.26E-12 | 4.04E-10 | -0.1446 | -0.06143 | -0.1014 |
| *g__norank_f__Erysipelotrichaceae* | 0.05887 | 0.1016 | 0.005963 | 0.04767 | 5.86E-12 | 4.04E-10 | -0.07939 | -0.02449 | -0.05291 |
| *g__Acinetobacter* | 0.0227 | 0.0383 | 0 | 0 | 1.55E-11 | 7.54E-10 | -0.03269 | -0.01379 | -0.02269 |
| *g__Rikenella* | 0.13 | 0.2198 | 9.46E-05 | 0.00054 | 2.08E-11 | 9.12E-10 | -0.1861 | -0.07877 | -0.1299 |
| *g__Anaeroplasma* | 0.02488 | 0.04518 | 0 | 0 | 3.87E-11 | 1.54E-09 | -0.03717 | -0.01437 | -0.02488 |
| *g__Corynebacterium_1* | 0.08766 | 0.1587 | 4.73E-05 | 0.000384 | 4.82E-11 | 1.76E-09 | -0.1335 | -0.04962 | -0.08761 |
| *g__Staphylococcus* | 0.1215 | 0.2101 | 0.000331 | 0.001655 | 6.53E-11 | 2.16E-09 | -0.1749 | -0.06763 | -0.1212 |
| *g__Prevotellaceae_UCG-001* | 0.06731 | 0.1096 | 0.003218 | 0.02427 | 6.91E-11 | 2.16E-09 | -0.09335 | -0.03634 | -0.06409 |
| *g__Acetatifactor* | 0.01619 | 0.02755 | 0 | 0 | 2.35E-10 | 6.42E-09 | -0.02405 | -0.00937 | -0.01619 |
| *g__Jeotgalicoccus* | 0.009838 | 0.01657 | 0 | 0 | 2.33E-10 | 6.42E-09 | -0.01416 | -0.00552 | -0.00984 |
| *g__norank_c__Cyanobacteria* | 0.08917 | 0.1476 | 0.001183 | 0.002436 | 4.6E-10 | 1.08E-08 | -0.1223 | -0.05186 | -0.08799 |
| *g__Lachnospiraceae_UCG-006* | 0.05434 | 0.09252 | 0.000284 | 0.001311 | 4.99E-10 | 1.08E-08 | -0.08023 | -0.03199 | -0.05406 |
| *g__Brevibacterium* | 0.03082 | 0.0563 | 0 | 0 | 5.65E-10 | 1.08E-08 | -0.04628 | -0.01754 | -0.03082 |
| *g__Dietzia* | 0.02759 | 0.04763 | 0 | 0 | 5.66E-10 | 1.08E-08 | -0.04008 | -0.01614 | -0.02759 |
| *g__Mucispirillum* | 0.01754 | 0.03161 | 0 | 0 | 5.66E-10 | 1.08E-08 | -0.02639 | -0.00999 | -0.01754 |
| *g__Treponema_2* | 0.01671 | 0.02854 | 9.46E-05 | 0.00054 | 4.38E-10 | 1.08E-08 | -0.02467 | -0.00922 | -0.01661 |
| *g__Sporosarcina* | 0.01411 | 0.02484 | 0 | 0 | 5.64E-10 | 1.08E-08 | -0.0204 | -0.00807 | -0.01411 |
| *g__Christensenella* | 0.05726 | 0.09743 | 0.000899 | 0.002834 | 1.16E-09 | 2.11E-08 | -0.08095 | -0.03197 | -0.05636 |
| *g__norank_c__Acidobacteria* | 0.01244 | 0.02274 | 4.73E-05 | 0.000384 | 2.06E-09 | 3.62E-08 | -0.01812 | -0.0072 | -0.01239 |
| *g__Ralstonia* | 0.01478 | 0.02707 | 0 | 0 | 3.15E-09 | 5.3E-08 | -0.02186 | -0.00797 | -0.01478 |
| *g__Enterorhabdus* | 0.02644 | 0.04349 | 0.000946 | 0.005849 | 3.33E-09 | 5.4E-08 | -0.03606 | -0.01504 | -0.0255 |

| | | | | | | | | | |
|---|---|---|---|---|---|---|---|---|---|
| *g__Bacillus* | 0.5491 | 4.068 | 0.002129 | 0.01249 | 6.85E-09 | 1.07E-07 | -1.602 | -0.01435 | -0.5471 |
| *g__unclassified_f__Family_XIII* | 0.01015 | 0.0187 | 0 | 0 | 7.27E-09 | 1.1E-07 | -0.01536 | -0.00583 | -0.01015 |
| *g__Pseudomonas* | 0.04982 | 0.1218 | 0.000189 | 0.000751 | 7.78E-09 | 1.14E-07 | -0.08252 | -0.02358 | -0.04962 |
| *g__RB41* | 0.01228 | 0.02039 | 4.73E-05 | 0.000384 | 1.14E-08 | 1.61E-07 | -0.01755 | -0.0074 | -0.01224 |
| *g__Atopostipes* | 0.01036 | 0.02109 | 0 | 0 | 1.67E-08 | 2.15E-07 | -0.01661 | -0.00515 | -0.01036 |
| *g__norank_f__Nitrosomonadaceae* | 0.006611 | 0.01335 | 0 | 0 | 1.66E-08 | 2.15E-07 | -0.01057 | -0.00328 | -0.00661 |
| *g__Shewanella* | 0.004893 | 0.008517 | 0 | 0 | 1.66E-08 | 2.15E-07 | -0.00729 | -0.00281 | -0.00489 |
| *g__norank_f__Clostridiales_vadinBB60_group* | 0.1909 | 0.3197 | 0.007761 | 0.02391 | 2.27E-08 | 2.84E-07 | -0.2675 | -0.1116 | -0.1832 |
| *g__Oscillibacter* | 0.08183 | 0.1367 | 0.005915 | 0.03308 | 2.92E-08 | 3.55E-07 | -0.1132 | -0.04164 | -0.07591 |
| *g__unclassified_f__Erysipelotrichaceae* | 0.06538 | 0.1133 | 0.003691 | 0.02209 | 3.41E-08 | 4.04E-07 | -0.09183 | -0.03328 | -0.06169 |
| *g__Rubrobacter* | 0.01202 | 0.02126 | 0 | 0 | 3.79E-08 | 4.25E-07 | -0.01765 | -0.00692 | -0.01202 |
| *g__Microvirga* | 0.005362 | 0.009363 | 0 | 0 | 3.77E-08 | 4.25E-07 | -0.00781 | -0.00323 | -0.00536 |
| *g__Nesterenkonia* | 0.01223 | 0.02255 | 4.73E-05 | 0.000384 | 6.35E-08 | 6.95E-07 | -0.01785 | -0.00699 | -0.01219 |
| *g__Paracoccus* | 0.008225 | 0.01564 | 4.73E-05 | 0.000384 | 6.57E-08 | 7.01E-07 | -0.0126 | -0.00463 | -0.00818 |
| *g__unclassified_o__Bacteroidales* | 0.05898 | 0.09907 | 0.002887 | 0.0102 | 8.14E-08 | 7.88E-07 | -0.08353 | -0.03209 | -0.05609 |
| *g__Nosocomiicoccus* | 0.007131 | 0.01306 | 0 | 0 | 8.45E-08 | 7.88E-07 | -0.01041 | -0.0039 | -0.00713 |
| *g__Micrococcus* | 0.00609 | 0.0111 | 0 | 0 | 8.45E-08 | 7.88E-07 | -0.00911 | -0.00333 | -0.00609 |
| *g__norank_f__ODP1230B8.23* | 0.004008 | 0.007411 | 0 | 0 | 8.36E-08 | 7.88E-07 | -0.00609 | -0.00219 | -0.00401 |
| *g__Achromobacter* | 0.002915 | 0.006969 | 0 | 0 | 8.22E-08 | 7.88E-07 | -0.00495 | -0.00146 | -0.00292 |
| *g__Bradyrhizobium* | 0.002394 | 0.004541 | 0 | 0 | 8.16E-08 | 7.88E-07 | -0.00354 | -0.00125 | -0.00239 |
| *g__Alloprevotella* | 2.616 | 4.159 | 1.127 | 4.466 | 9.44E-08 | 8.62E-07 | -2.858 | 0.06179 | -1.489 |
| *g__norank_f__Peptococcaceae* | 0.03685 | 0.06148 | 0.002934 | 0.0146 | 9.64E-08 | 8.62E-07 | -0.05008 | -0.01877 | -0.03392 |
| *g__Ruminiclostridium* | 0.02946 | 0.05136 | 0.000852 | 0.003125 | 1.35E-07 | 1.18E-06 | -0.042 | -0.01648 | -0.02861 |
| *g__Rhizobium* | 0.006507 | 0.01213 | 9.46E-05 | 0.000769 | 1.83E-07 | 1.44E-06 | -0.00944 | -0.00359 | -0.00641 |
| *g__Brachybacterium* | 0.005934 | 0.01052 | 0 | 0 | 1.87E-07 | 1.44E-06 | -0.00859 | -0.00344 | -0.00593 |
| *g__norank_f__Acidobacteriaceae__Subgroup_1_* | 0.004529 | 0.008242 | 0 | 0 | 1.86E-07 | 1.44E-06 | -0.00677 | -0.0026 | -0.00453 |
| *g__Stenotrophomonas* | 0.004164 | 0.00749 | 0 | 0 | 1.86E-07 | 1.44E-06 | -0.00604 | -0.00239 | -0.00416 |
| *g__Mizugakiibacter* | 0.00406 | 0.008574 | 0 | 0 | 1.86E-07 | 1.44E-06 | -0.00625 | -0.00219 | -0.00406 |
| *g__Burkholderia-Paraburkholderia* | 0.0038 | 0.007321 | 0 | 0 | 1.86E-07 | 1.44E-06 | -0.00593 | -0.00208 | -0.0038 |
| *g__norank_f__Xanthobacteraceae* | 0.00354 | 0.007375 | 0 | 0 | 1.85E-07 | 1.44E-06 | -0.00536 | -0.00193 | -0.00354 |
| *g__[Eubacterium]_nodatum_group* | 0.02764 | 0.04152 | 0.004259 | 0.01344 | 2.16E-07 | 1.63E-06 | -0.03385 | -0.01272 | -0.02338 |

| | | | | | | | | | |
|---|---|---|---|---|---|---|---|---|---|
| *g__Enterococcus* | 0.6214 | 1.04 | 0.05314 | 0.3338 | 3.14E-07 | 2.33E-06 | -0.8427 | -0.3006 | -0.5682 |
| *g__norank_o__SC-I-84* | 0.00432 | 0.008114 | 4.73E-05 | 0.000384 | 3.34E-07 | 2.44E-06 | -0.00662 | -0.00246 | -0.00427 |
| *g__norank_f__288-2* | 0.004737 | 0.008843 | 0 | 0 | 4.08E-07 | 2.75E-06 | -0.00708 | -0.0026 | -0.00474 |
| *g__Flavobacterium* | 0.003384 | 0.006437 | 0 | 0 | 4.05E-07 | 2.75E-06 | -0.0051 | -0.00193 | -0.00338 |
| *g__norank_c__KD4-96* | 0.002915 | 0.005359 | 0 | 0 | 4.07E-07 | 2.75E-06 | -0.00427 | -0.00167 | -0.00292 |
| *g__unclassified_f__Xanthobacteraceae* | 0.002655 | 0.00504 | 0 | 0 | 4.04E-07 | 2.75E-06 | -0.00401 | -0.00146 | -0.00266 |
| *g__Anaerovorax* | 0.00177 | 0.003063 | 0 | 0 | 3.99E-07 | 2.75E-06 | -0.0025 | -0.00104 | -0.00177 |
| *g__norank_f__Rhodospirillaceae* | 0.03019 | 0.06756 | 0.008613 | 0.03954 | 5.78E-07 | 3.84E-06 | -0.04562 | -0.00434 | -0.02158 |
| *g__Sphingomonas* | 0.003748 | 0.007246 | 4.73E-05 | 0.000384 | 7.49E-07 | 4.89E-06 | -0.00563 | -0.00198 | -0.0037 |
| *g__unclassified_f__Comamonadaceae* | 0.00531 | 0.01064 | 0 | 0 | 8.83E-07 | 5.3E-06 | -0.00812 | -0.00276 | -0.00531 |
| *g__Blastococcus* | 0.003956 | 0.00756 | 0 | 0 | 8.83E-07 | 5.3E-06 | -0.00593 | -0.00229 | -0.00396 |
| *g__Vibrio* | 0.003956 | 0.008775 | 0 | 0 | 8.81E-07 | 5.3E-06 | -0.00666 | -0.00198 | -0.00396 |
| *g__norank_p__WS6* | 0.00354 | 0.006767 | 0 | 0 | 8.82E-07 | 5.3E-06 | -0.00526 | -0.00198 | -0.00354 |
| *g__Soonwooa* | 0.003071 | 0.005793 | 0 | 0 | 8.82E-07 | 5.3E-06 | -0.00458 | -0.00161 | -0.00307 |
| *g__Pseudonocardia* | 0.001926 | 0.003641 | 0 | 0 | 8.66E-07 | 5.3E-06 | -0.00292 | -0.00109 | -0.00193 |
| *g__Enterobacter* | 0.9451 | 2.834 | 0.8529 | 3.033 | 9.73E-07 | 5.76E-06 | -1.151 | 0.9806 | -0.09228 |
| *g__Ruminococcaceae_UCG-014* | 0.7164 | 1.065 | 0.2025 | 0.4618 | 1.44E-06 | 8.44E-06 | -0.8068 | -0.2496 | -0.5139 |
| *g__norank_f__Christensenellaceae* | 0.01728 | 0.04355 | 0.002129 | 0.005645 | 1.73E-06 | 9.95E-06 | -0.02696 | -0.00684 | -0.01515 |
| *g__Halanaerobium* | 0.004841 | 0.01025 | 0 | 0 | 1.89E-06 | 1.01E-05 | -0.00755 | -0.0024 | -0.00484 |
| *g__norank_o__Gastranaerophilales* | 0.00406 | 0.008118 | 0 | 0 | 1.89E-06 | 1.01E-05 | -0.00619 | -0.00224 | -0.00406 |
| *g__Aeromonas* | 0.003904 | 0.01442 | 0 | 0 | 1.89E-06 | 1.01E-05 | -0.00848 | -0.00141 | -0.0039 |
| *g__norank_c__Actinobacteria* | 0.002394 | 0.004892 | 0 | 0 | 1.88E-06 | 1.01E-05 | -0.00364 | -0.0013 | -0.00239 |
| *g__Arthrobacter* | 0.002394 | 0.004613 | 0 | 0 | 1.88E-06 | 1.01E-05 | -0.00349 | -0.0013 | -0.00239 |
| *g__norank_p__Tectomicrobia* | 0.001666 | 0.00352 | 0 | 0 | 1.86E-06 | 1.01E-05 | -0.00271 | -0.00088 | -0.00167 |
| *g__Akkermansia* | 0.2031 | 0.8314 | 0.05702 | 0.2295 | 1.93E-06 | 1.02E-05 | -0.3736 | 0.01884 | -0.1461 |
| *g__Lactobacillus* | 3.5 | 6.035 | 0.6953 | 3.346 | 2.69E-06 | 0.000014 | -4.535 | -0.9814 | -2.805 |
| *g__Catabacter* | 0.004841 | 0.008479 | 0.000284 | 0.001311 | 2.85E-06 | 1.47E-05 | -0.00693 | -0.00263 | -0.00456 |
| *g__Odoribacter* | 0.7093 | 0.9294 | 0.1544 | 0.251 | 3.26E-06 | 1.66E-05 | -0.8042 | -0.3107 | -0.5549 |
| *g__Solirubrobacter* | 0.002967 | 0.006336 | 4.73E-05 | 0.000384 | 3.55E-06 | 1.79E-05 | -0.00453 | -0.00142 | -0.00292 |
| *g__Arcobacter* | 0.00203 | 0.003674 | 4.73E-05 | 0.000384 | 3.74E-06 | 1.86E-05 | -0.00287 | -0.00109 | -0.00198 |

| | | | | | | | | | |
|---|---|---|---|---|---|---|---|---|---|
| *g__Lysinibacillus* | 0.01431 | 0.09297 | 0 | 0 | 4E-06 | 1.87E-05 | -0.04893 | -0.00141 | -0.01432 |
| *g__unclassified_f__Micromonosporaceae* | 0.005205 | 0.01119 | 0 | 0 | 4.01E-06 | 1.87E-05 | -0.00797 | -0.00271 | -0.00521 |
| *g__norank_o__Gaiellales* | 0.004945 | 0.009747 | 0 | 0 | 4.01E-06 | 1.87E-05 | -0.0076 | -0.00281 | -0.00495 |
| *g__norank_o__JG30-KF-CM45* | 0.003279 | 0.007006 | 0 | 0 | 4E-06 | 1.87E-05 | -0.00526 | -0.00161 | -0.00328 |
| *g__norank_o__AKYG1722* | 0.002238 | 0.004383 | 0 | 0 | 3.99E-06 | 1.87E-05 | -0.00349 | -0.00115 | -0.00224 |
| *g__norank_f__Gemmatimonadaceae* | 0.002082 | 0.004093 | 0 | 0 | 3.99E-06 | 1.87E-05 | -0.00318 | -0.00115 | -0.00208 |
| *g__norank_f__Flavobacteriaceae* | 0.00229 | 0.004909 | 9.46E-05 | 0.000769 | 4.93E-06 | 2.27E-05 | -0.0037 | -0.00109 | -0.0022 |
| *g__Morganella* | 0.001978 | 0.005269 | 9.46E-05 | 0.000769 | 5.42E-06 | 2.48E-05 | -0.00345 | -0.00081 | -0.00188 |
| *g__norank_c__Gitt-GS-136* | 0.002915 | 0.006502 | 0 | 0 | 8.39E-06 | 3.68E-05 | -0.00468 | -0.00135 | -0.00292 |
| *g__Nitrospira* | 0.002707 | 0.006232 | 0 | 0 | 8.37E-06 | 3.68E-05 | -0.00427 | -0.0013 | -0.00271 |
| *g__Sphingobium* | 0.00229 | 0.004523 | 0 | 0 | 8.4E-06 | 3.68E-05 | -0.00338 | -0.0012 | -0.00229 |
| *g__Acidibacter* | 0.001458 | 0.002786 | 0 | 0 | 8.34E-06 | 3.68E-05 | -0.00219 | -0.00083 | -0.00146 |
| *g__Mycobacterium* | 0.002499 | 0.004409 | 0.000142 | 0.000656 | 9.95E-06 | 4.32E-05 | -0.00351 | -0.00131 | -0.00236 |
| *g__Citrobacter* | 0.3332 | 1.795 | 0.02896 | 0.08211 | 1.32E-05 | 5.67E-05 | -0.7937 | -0.02906 | -0.3042 |
| *g__Lactococcus* | 0.03352 | 0.09106 | 0.001514 | 0.006156 | 1.65E-05 | 7.02E-05 | -0.05708 | -0.0114 | -0.03201 |
| *g__Caulobacter* | 0.002915 | 0.0111 | 0 | 0 | 1.74E-05 | 7.07E-05 | -0.00599 | -0.00094 | -0.00292 |
| *g__Geodermatophilus* | 0.002447 | 0.005518 | 0 | 0 | 1.74E-05 | 7.07E-05 | -0.00396 | -0.0013 | -0.00245 |
| *g__Thermus* | 0.001822 | 0.00374 | 0 | 0 | 1.74E-05 | 7.07E-05 | -0.00292 | -0.00094 | -0.00182 |
| *g__norank_f__Rhodobiaceae* | 0.00177 | 0.00404 | 0 | 0 | 1.74E-05 | 7.07E-05 | -0.00292 | -0.00088 | -0.00177 |
| *g__Streptomyces* | 0.001562 | 0.003227 | 0 | 0 | 1.74E-05 | 7.07E-05 | -0.00245 | -0.00083 | -0.00156 |
| *g__Proteus* | 0.002863 | 0.00512 | 0.000189 | 0.000751 | 2.35E-05 | 9.42E-05 | -0.00413 | -0.00142 | -0.00267 |
| *g__Alcaligenes* | 0.007496 | 0.04669 | 0 | 0 | 3.59E-05 | 0.000134 | -0.02488 | -0.00088 | -0.0075 |
| *g__Dongia* | 0.002394 | 0.007028 | 0 | 0 | 3.58E-05 | 0.000134 | -0.00432 | -0.00078 | -0.00239 |
| *g__H16* | 0.001874 | 0.004424 | 0 | 0 | 3.59E-05 | 0.000134 | -0.00307 | -0.00088 | -0.00187 |
| *g__Microlunatus* | 0.001614 | 0.003792 | 0 | 0 | 3.57E-05 | 0.000134 | -0.00266 | -0.00078 | -0.00161 |
| *g__Elizabethkingia* | 0.001353 | 0.002953 | 0 | 0 | 3.58E-05 | 0.000134 | -0.00213 | -0.00068 | -0.00135 |
| *g__Truepera* | 0.001249 | 0.002648 | 0 | 0 | 3.57E-05 | 0.000134 | -0.00193 | -0.00057 | -0.00125 |
| *g__norank_f__P-102* | 0.001197 | 0.002703 | 0 | 0 | 3.57E-05 | 0.000134 | -0.00198 | -0.00057 | -0.0012 |
| *g__Roseiflexus* | 0.001093 | 0.00256 | 0 | 0 | 3.53E-05 | 0.000134 | -0.00172 | -0.00052 | -0.00109 |
| *g__Parvibacter* | 0.002603 | 0.00446 | 0.000142 | 0.000656 | 3.65E-05 | 0.000135 | -0.00377 | -0.00134 | -0.00246 |
| *g__Klebsiella* | 0.5667 | 1.063 | 0.3449 | 1.053 | 3.78E-05 | 0.000139 | -0.5928 | 0.1807 | -0.2218 |

| | | | | | | | | | |
|---|---|---|---|---|---|---|---|---|---|
| *g__norank_f__Bacteroidales_S24-7_group* | 4.856 | 7.436 | 0.6246 | 1.416 | 3.83E-05 | 0.00014 | -6.239 | -2.455 | -4.231 |
| *g__unclassified_f__Prevotellaceae* | 0.00203 | 0.005138 | 9.46E-05 | 0.00054 | 6.52E-05 | 0.000236 | -0.00339 | -0.00085 | -0.00194 |
| *g__Cloacibacterium* | 0.003071 | 0.01241 | 0 | 0 | 7.33E-05 | 0.000253 | -0.00661 | -0.00083 | -0.00307 |
| *g__Gaiella* | 0.00229 | 0.005359 | 4.73E-05 | 0.000384 | 7.2E-05 | 0.000253 | -0.0037 | -0.00095 | -0.00224 |
| *g__Psychroglaciecola* | 0.001718 | 0.004083 | 0 | 0 | 7.32E-05 | 0.000253 | -0.00292 | -0.00078 | -0.00172 |
| *g__unclassified_f__Archangiaceae* | 0.001301 | 0.003059 | 0 | 0 | 7.31E-05 | 0.000253 | -0.00213 | -0.00057 | -0.0013 |
| *g__Modestobacter* | 0.001093 | 0.002359 | 0 | 0 | 7.3E-05 | 0.000253 | -0.00177 | -0.00052 | -0.00109 |
| *g__norank_f__DA101_soil_group* | 0.001041 | 0.002276 | 0 | 0 | 7.29E-05 | 0.000253 | -0.00161 | -0.00052 | -0.00104 |
| *g__unclassified_f__Ruminococcaceae* | 0.4013 | 0.6113 | 0.0566 | 0.07884 | 7.46E-05 | 0.000255 | -0.5108 | -0.191 | -0.3448 |
| *g__unclassified_f__Enterobacteriaceae* | 0.1591 | 0.8805 | 0.02262 | 0.1006 | 1E-04 | 0.00034 | -0.3823 | 0.01005 | -0.1365 |
| *g__Brevundimonas* | 0.006507 | 0.03949 | 0 | 0 | 0.000148 | 0.000475 | -0.01697 | -0.00078 | -0.00651 |
| *g__norank_f__JG34-KF-361* | 0.001562 | 0.00366 | 0 | 0 | 0.000148 | 0.000475 | -0.00255 | -0.00068 | -0.00156 |
| *g__Leucobacter* | 0.001405 | 0.003325 | 0 | 0 | 0.000148 | 0.000475 | -0.00234 | -0.00057 | -0.00141 |
| *g__Arenimonas* | 0.001353 | 0.003371 | 0 | 0 | 0.000148 | 0.000475 | -0.00229 | -0.00057 | -0.00135 |
| *g__norank_f__Planctomycetaceae* | 0.001353 | 0.003271 | 0 | 0 | 0.000148 | 0.000475 | -0.00224 | -0.00057 | -0.00135 |
| *g__Gemmatimonas* | 0.001145 | 0.002504 | 0 | 0 | 0.000148 | 0.000475 | -0.00177 | -0.00057 | -0.00115 |
| *g__norank_o__Xanthomonadales* | 0.000833 | 0.001896 | 0 | 0 | 0.000147 | 0.000475 | -0.00135 | -0.00036 | -0.00083 |
| *g__Bryobacter* | 0.000781 | 0.001689 | 0 | 0 | 0.000147 | 0.000475 | -0.00125 | -0.00036 | -0.00078 |
| *g__norank_o__Mollicutes_RF9* | 0.1368 | 0.4176 | 0.03805 | 0.1002 | 0.000155 | 0.000491 | -0.2364 | -0.01591 | -0.09875 |
| *g__Massilia* | 0.001353 | 0.003008 | 4.73E-05 | 0.000384 | 0.000157 | 0.000495 | -0.00219 | -0.00058 | -0.00131 |
| *g__[Eubacterium]_fissicatena_group* | 0.08349 | 0.1296 | 0.01287 | 0.02467 | 0.000231 | 0.000724 | -0.1072 | -0.03874 | -0.07063 |
| *g__Conexibacter* | 0.001614 | 0.00599 | 0 | 0 | 0.000297 | 0.000887 | -0.00333 | -0.00042 | -0.00161 |
| *g__Steroidobacter* | 0.001145 | 0.003041 | 0 | 0 | 0.000298 | 0.000887 | -0.00198 | -0.00047 | -0.00115 |
| *g__Virgisporangium* | 0.001145 | 0.002815 | 0 | 0 | 0.000298 | 0.000887 | -0.00187 | -0.00047 | -0.00115 |
| *g__Blastocatella* | 0.000885 | 0.002081 | 0 | 0 | 0.000297 | 0.000887 | -0.00146 | -0.00036 | -0.00088 |
| *g__norank_f__Elev-16S-1332* | 0.000885 | 0.002081 | 0 | 0 | 0.000297 | 0.000887 | -0.00146 | -0.00042 | -0.00088 |
| *g__Rhodoplanes* | 0.000885 | 0.002081 | 0 | 0 | 0.000297 | 0.000887 | -0.00141 | -0.00042 | -0.00088 |
| *g__Skermanella* | 0.000833 | 0.001896 | 0 | 0 | 0.000297 | 0.000887 | -0.00135 | -0.00036 | -0.00083 |
| *g__unclassified_o__Clostridiales* | 0.01869 | 0.02562 | 0.006862 | 0.01443 | 0.000373 | 0.001102 | -0.01941 | -0.0041 | -0.01183 |
| *g__Family_XIII_AD3011_group* | 0.0393 | 0.04509 | 0.01547 | 0.0229 | 0.000544 | 0.001598 | -0.03636 | -0.01212 | -0.02383 |

| | | | | | | | | | |
|---|---|---|---|---|---|---|---|---|---|
| *g__Pseudoalteromonas* | 0.001718 | 0.004433 | 0 | 0 | 0.000594 | 0.001658 | -0.00292 | -0.00062 | -0.00172 |
| *g__norank_o__C0119* | 0.000989 | 0.002603 | 0 | 0 | 0.000594 | 0.001658 | -0.00167 | -0.00036 | -0.00099 |
| *g__unclassified_f__Acidobacteriaceae__Subgroup_1_* | 0.000989 | 0.002666 | 0 | 0 | 0.000594 | 0.001658 | -0.00172 | -0.00036 | -0.00099 |
| *g__Nitrosomonas* | 0.000937 | 0.002654 | 0 | 0 | 0.000593 | 0.001658 | -0.00167 | -0.00036 | -0.00094 |
| *g__Chryseobacterium* | 0.000885 | 0.002159 | 0 | 0 | 0.000594 | 0.001658 | -0.00146 | -0.00036 | -0.00088 |
| *g__Sulfurimonas* | 0.000781 | 0.001961 | 0 | 0 | 0.000593 | 0.001658 | -0.00135 | -0.00031 | -0.00078 |
| *g__norank_f__Acidimicrobiaceae* | 0.000781 | 0.002043 | 0 | 0 | 0.000592 | 0.001658 | -0.0013 | -0.00031 | -0.00078 |
| *g__Pelomonas* | 0.000729 | 0.001851 | 0 | 0 | 0.000592 | 0.001658 | -0.00125 | -0.00026 | -0.00073 |
| *g__Desulfovibrio* | 0.5013 | 0.7871 | 0.1393 | 0.3278 | 0.000624 | 0.00173 | -0.5794 | -0.156 | -0.362 |
| *g__Flavonifractor* | 0.06611 | 0.125 | 0.1725 | 0.3951 | 0.000628 | 0.00173 | 0.01691 | 0.2147 | 0.1064 |
| *g__[Eubacterium]_xylanophilum_group* | 0.0431 | 0.09006 | 0.04604 | 0.1468 | 0.00098 | 0.002683 | -0.03574 | 0.0507 | 0.002945 |
| *g__Eisenbergiella* | 0.01228 | 0.02171 | 0.01126 | 0.04366 | 0.001009 | 0.002745 | -0.01178 | 0.01252 | -0.00102 |
| *g__[Eubacterium]_brachy_group* | 0.01994 | 0.03171 | 0.01169 | 0.06651 | 0.001105 | 0.002987 | -0.02279 | 0.01266 | -0.00825 |
| *g__Tissierella* | 0.01145 | 0.08502 | 0 | 0 | 0.001174 | 0.003102 | -0.03363 | -0.00026 | -0.01145 |
| *g__unclassified_f__Xanthomonadaceae* | 0.000729 | 0.002102 | 0 | 0 | 0.001176 | 0.003102 | -0.00135 | -0.00026 | -0.00073 |
| *g__unclassified_c__Betaproteobacteria* | 0.000677 | 0.001915 | 0 | 0 | 0.001174 | 0.003102 | -0.0012 | -0.00026 | -0.00068 |
| *g__Kurthia* | 0.000573 | 0.001465 | 0 | 0 | 0.001174 | 0.003102 | -0.00094 | -0.00021 | -0.00057 |
| *g__Weissella* | 0.01994 | 0.06107 | 0.000473 | 0.002139 | 0.001267 | 0.003303 | -0.03699 | -0.00609 | -0.01946 |
| *g__Propionibacterium* | 0.001093 | 0.002624 | 4.73E-05 | 0.000384 | 0.001267 | 0.003303 | -0.00177 | -0.00043 | -0.00105 |
| *g__norank_f__Lachnospiraceae* | 1.022 | 0.9018 | 0.7916 | 1.435 | 0.001629 | 0.004221 | -0.6233 | 0.217 | -0.2307 |
| *g__Comamonas* | 0.001405 | 0.003833 | 9.46E-05 | 0.00054 | 0.002286 | 0.005682 | -0.00231 | -0.00048 | -0.00131 |
| *g__norank_c__S085* | 0.000677 | 0.001915 | 0 | 0 | 0.002322 | 0.005682 | -0.0012 | -0.00026 | -0.00068 |
| *g__Azospirillum* | 0.000677 | 0.002578 | 0 | 0 | 0.002314 | 0.005682 | -0.00141 | -0.00021 | -0.00068 |
| *g__Aquabacterium* | 0.000625 | 0.0018 | 0 | 0 | 0.002321 | 0.005682 | -0.00109 | -0.00026 | -0.00062 |
| *g__Sphingobacterium* | 0.000573 | 0.001863 | 0 | 0 | 0.002314 | 0.005682 | -0.00109 | -0.00021 | -0.00057 |
| *g__Actinophytocola* | 0.000573 | 0.001676 | 0 | 0 | 0.002318 | 0.005682 | -0.00104 | -0.00021 | -0.00057 |
| *g__Taibaiella* | 0.000573 | 0.001676 | 0 | 0 | 0.002318 | 0.005682 | -0.00104 | -0.00021 | -0.00057 |
| *g__Venenivibrio* | 0.000573 | 0.001574 | 0 | 0 | 0.00232 | 0.005682 | -0.00099 | -0.00021 | -0.00057 |
| *g__unclassified_f__Intrasporangiaceae* | 0.000521 | 0.001539 | 0 | 0 | 0.002314 | 0.005682 | -0.00094 | -0.00021 | -0.00052 |
| *g__Nordella* | 0.000469 | 0.001263 | 0 | 0 | 0.002314 | 0.005682 | -0.00083 | -0.00016 | -0.00047 |
| *g__Ruminococcaceae_UCG-009* | 0.02868 | 0.03656 | 0.01358 | 0.0245 | 0.003183 | 0.007744 | -0.02584 | -0.00496 | -0.0151 |

| | | | | | | | | | |
|---|---|---|---|---|---|---|---|---|---|
| *g__unclassified_f__Christensenellaceae* | 0.002082 | 0.004173 | 0.000994 | 0.004319 | 0.003444 | 0.008333 | -0.00248 | 0.000516 | -0.00109 |
| *g__Coprococcus_1* | 0.121 | 0.1397 | 0.07169 | 0.08931 | 0.004159 | 0.01001 | -0.09582 | -0.01011 | -0.04933 |
| *g__Pedobacter* | 0.000677 | 0.002234 | 0 | 0 | 0.00456 | 0.01057 | -0.0013 | -0.00016 | -0.00068 |
| *g__Lechevalieria* | 0.000625 | 0.002057 | 0 | 0 | 0.00456 | 0.01057 | -0.0012 | -0.00021 | -0.00062 |
| *g__Ureaplasma* | 0.000573 | 0.001772 | 0 | 0 | 0.00456 | 0.01057 | -0.00104 | -0.00021 | -0.00057 |
| *g__Luteibacter* | 0.000469 | 0.001388 | 0 | 0 | 0.004556 | 0.01057 | -0.00083 | -0.00016 | -0.00047 |
| *g__Reyranella* | 0.000469 | 0.001502 | 0 | 0 | 0.00455 | 0.01057 | -0.00088 | -0.00016 | -0.00047 |
| *g__Tumebacillus* | 0.000469 | 0.001388 | 0 | 0 | 0.004556 | 0.01057 | -0.00083 | -0.00016 | -0.00047 |
| *g__unclassified_f__Erythrobacteraceae* | 0.000364 | 0.001011 | 0 | 0 | 0.004542 | 0.01057 | -0.00062 | -0.00016 | -0.00036 |
| *g__Ruminococcaceae_UCG-010* | 0.04039 | 0.07559 | 0.04519 | 0.1507 | 0.005119 | 0.0118 | -0.03301 | 0.05252 | 0.004802 |
| *g__norank_p__Saccharibacteria* | 0.02499 | 0.04291 | 0.0151 | 0.02981 | 0.005453 | 0.01251 | -0.02326 | 0.002943 | -0.00989 |
| *g__Clostridium_sensu_stricto_3* | 0.000573 | 0.001574 | 9.46E-05 | 0.000769 | 0.0061 | 0.01392 | -0.00094 | -6.2E-05 | -0.00048 |
| *g__unclassified_f__Coriobacteriaceae* | 0.001874 | 0.004535 | 0.000615 | 0.002206 | 0.007476 | 0.01697 | -0.0027 | -9E-05 | -0.00126 |
| *g__Tyzzerella* | 0.01291 | 0.02781 | 0.02238 | 0.09932 | 0.007977 | 0.01801 | -0.01162 | 0.03959 | 0.009472 |
| *g__Sellimonas* | 0.001093 | 0.003629 | 0.01978 | 0.06932 | 0.008081 | 0.01815 | 0.005309 | 0.03754 | 0.01869 |
| *g__Nocardioides* | 0.001041 | 0.005353 | 0 | 0 | 0.008935 | 0.01938 | -0.0026 | -0.0001 | -0.00104 |
| *g__norank_f__Caulobacteraceae* | 0.000677 | 0.002513 | 0 | 0 | 0.008936 | 0.01938 | -0.00141 | -0.00016 | -0.00068 |
| *g__norank_f__Mitochondria* | 0.000416 | 0.001344 | 0 | 0 | 0.00893 | 0.01938 | -0.00078 | -0.0001 | -0.00042 |
| *g__Mycoplasma* | 0.000416 | 0.001344 | 0 | 0 | 0.00893 | 0.01938 | -0.00083 | -0.00016 | -0.00042 |
| *g__norank_o__JG30-KF-AS9* | 0.000416 | 0.001344 | 0 | 0 | 0.00893 | 0.01938 | -0.00078 | -0.0001 | -0.00042 |
| *g__Vitreoscilla* | 0.000364 | 0.001163 | 0 | 0 | 0.008924 | 0.01938 | -0.00068 | -0.0001 | -0.00036 |
| *g__Rhizomicrobium* | 0.000364 | 0.001163 | 0 | 0 | 0.008924 | 0.01938 | -0.00073 | -0.0001 | -0.00036 |
| *g__Parasutterella* | 0.2367 | 0.4548 | 0.3006 | 0.8893 | 0.009103 | 0.01964 | -0.1663 | 0.3284 | 0.0639 |
| *g__Solobacterium* | 0.004633 | 0.01242 | 0.001893 | 0.005018 | 0.01003 | 0.02152 | -0.00663 | 0.000142 | -0.00274 |
| *g__Candidatus_Solibacter* | 0.000625 | 0.0018 | 4.73E-05 | 0.000384 | 0.01012 | 0.02152 | -0.00109 | -0.00016 | -0.00058 |
| *g__Pedomicrobium* | 0.000625 | 0.0018 | 4.73E-05 | 0.000384 | 0.01012 | 0.02152 | -0.00105 | -0.00018 | -0.00058 |
| *g__Bacteroides* | 13.68 | 11.73 | 21.89 | 17.64 | 0.01041 | 0.02202 | 2.895 | 13.08 | 8.208 |
| *g__Peptococcus* | 0.006975 | 0.01105 | 0.006909 | 0.02533 | 0.01194 | 0.02515 | -0.00599 | 0.007545 | -6.7E-05 |
| *g__Escherichia-Shigella* | 1.686 | 3.634 | 1.949 | 5.607 | 0.01497 | 0.03137 | -1.291 | 2.003 | 0.2625 |
| *g__unclassified_f__Rhodobacteraceae* | 0.000416 | 0.001571 | 0 | 0 | 0.0175 | 0.03501 | -0.00083 | -0.0001 | -0.00042 |

| | | | | | | | | | |
|---|---|---|---|---|---|---|---|---|---|
| *g__norank_d__Bacteria* | 0.000364 | 0.001419 | 0 | 0 | 0.0175 | 0.03501 | -0.00078 | -5.2E-05 | -0.00036 |
| *g__Craurococcus* | 0.000364 | 0.001419 | 0 | 0 | 0.0175 | 0.03501 | -0.00078 | -5.2E-05 | -0.00036 |
| *g__norank_f__Chitinophagaceae* | 0.000364 | 0.001298 | 0 | 0 | 0.0175 | 0.03501 | -0.00073 | -5.2E-05 | -0.00036 |
| *g__unclassified_o__Acidimicrobiales* | 0.000312 | 0.001106 | 0 | 0 | 0.0175 | 0.03501 | -0.00062 | -5.2E-05 | -0.00031 |
| *g__norank_f__KF-JG30-B3* | 0.000312 | 0.001106 | 0 | 0 | 0.0175 | 0.03501 | -0.00062 | -5.2E-05 | -0.00031 |
| *g__norank_c__JG30-KF-CM66* | 0.00026 | 0.000871 | 0 | 0 | 0.01748 | 0.03501 | -0.00052 | -5.2E-05 | -0.00026 |
| *g__norank_f__FFCH7168* | 0.00026 | 0.000871 | 0 | 0 | 0.01748 | 0.03501 | -0.00052 | -5.2E-05 | -0.00026 |
| *g__Candidatus_Arthromitus* | 0.00026 | 0.000871 | 0 | 0 | 0.01748 | 0.03501 | -0.00052 | -5.2E-05 | -0.00026 |
| *g__Pirellula* | 0.00026 | 0.000871 | 0 | 0 | 0.01748 | 0.03501 | -0.00047 | -5.2E-05 | -0.00026 |
| *g__Oscillospira* | 0.000364 | 0.001734 | 0.001514 | 0.003883 | 0.01839 | 0.0366 | 0.00018 | 0.002224 | 0.00115 |
| *g__Gordonibacter* | 0.002447 | 0.005114 | 0.001514 | 0.005485 | 0.02231 | 0.04422 | -0.00272 | 0.001135 | -0.00093 |
